# How to activate intuitive and reflective thinking in behavior research? A comprehensive examination of experimental techniques

**DOI:** 10.3758/s13428-022-01984-4

**Published:** 2022-10-17

**Authors:** Ozan Isler, Onurcan Yilmaz

**Affiliations:** 1 School of Economics, University of Queensland, St Lucia, Australia; 2 Department of Psychology, Kadir Has University, Istanbul, Turkey

**Keywords:** Intuition, Reflection, Debiasing training, Induction, Recall, Time limits, Justification, Cognitive load, Monetary incentives

## Abstract

Experiments comparing intuitive and reflective decisions provide insights into the cognitive foundations of human behavior. However, the relative strengths and weaknesses of the frequently used experimental techniques for activating intuition and reflection remain unknown. In a large-scale preregistered online experiment (*N* = 3667), we compared the effects of eight reflection, six intuition, and two within-subjects manipulations on actual and self-reported measures of cognitive performance. Compared to the overall control, the long debiasing training was the most effective technique for increasing actual reflection scores, and the emotion induction was the most effective technique for increasing actual intuition scores. In contrast, the reason and the intuition recall, the reason induction, and the brief time delay conditions failed to achieve the intended effects. We recommend using the debiasing training, the decision justification, or the monetary incentives technique to activate reflection, and the emotion induction, the cognitive load, or the time pressure technique to activate intuition.

**Supplementary Information:**

The online version contains supplementary material available at 10.3758/s13428-022-01984-4.

## Introduction

Intuition and reflection are fundamental concepts in modern behavior research, referring to fast, automatic, and low-effort thinking on the one hand, or to slow, controlled, and high-effort thinking on the other (Kahneman, 2011). Standard dual-process models of the mind assume a cognitive hierarchy where reflection overrides and corrects intuition (Evans & Stanovich, 2013; Morewedge & Kahneman, 2010), whereas more recent theories view the relationship between the two as more complex (Bago & De Neys, 2017; De Neys, 2021; De Neys & Pennycook, 2019; Krajbich et al., 2015; Pennycook et al., 2015; Teoh et al., 2020; Thompson et al., 2009). Experiments manipulating decisions to be relatively more intuitive or reflective have helped to advance these theoretical debates, allowing causal insights into wide-ranging phenomena in the fields of judgment and decision-making, moral dilemmas, political ideology, religious belief, and social behavior (e.g., Baron et al., 2015; Białek & De Neys, 2016; Gervais et al., 2018; Isler et al., 2018; Isler, Gächter, Maule, & Starmer, 2021a; Isler & Yilmaz, 2019; Isler, Yilmaz, & Maule, 2021c; Nurse et al., 2021; Pennycook et al., 2012; Pennycook et al., 2014; Rand et al., 2012; Swami et al., 2014; Trémolière et al., 2014; Yilmaz, 2021; Yilmaz & Isler, 2019; Yilmaz & Saribay, 2017a, 2017b).

However, previous experimental research that has manipulated intuition and reflection suffers from five main methodological problems. First, recent tests indicate that some of these techniques often fail to effectively manipulate thinking styles (e.g., Deppe et al., 2015; Isler et al., 2020; Yilmaz & Saribay, 2016). Second, an increasing number of high-powered replication failures suggests that many of the previously identified effects could have been artifacts of methodological weaknesses such as small sample size (e.g., O’Donnell et al., 2021). Third, it is often difficult to make systematic comparisons between previous experimental findings due to the heterogeneity of techniques used in manipulating intuition and reflection. Fourth, manipulation checks for testing method effectiveness often rest on participants’ own evaluations, but these self-report measures can be unreliable because “direct introspective access to higher order cognitive processes” can be limited (Nisbett & Wilson, 1977) and because self-report data can be influenced by desirable responding (Furnham, 1986; Holtgraves, 2004). Finally, because these experimental manipulations were originally developed and tested in the laboratory, it is difficult to use them in online studies, which have become more common in behavior research (e.g., Peyton et al., 2021).

The present study aims to overcome these problems by providing the first systematic experimental comparison of a comprehensive set of promising intuition and reflection manipulations in a large-scale online experiment using performance measures. Only two previous studies have attempted to provide such systematic comparisons of thinking style manipulations: Isler et al. (2020), who focused exclusively on reflection manipulations, and Deck et al. (2017), who solely considered intuition manipulations. Since intuition and reflection manipulations are often used concurrently (e.g., time pressure vs. delay; emotion vs. reason induction), lack of an experimental comparison that simultaneously incorporates both types of manipulations means that the reliability of techniques for activating reflection and intuition remains relatively unknown.

We employed eight reflection and six intuition manipulations using a between-subjects design. In addition, we employed two different versions of the two-response elicitation technique that elicits an initial answer under time pressure and a final answer after a time delay using a within-subjects design. The effects of these manipulations on actual and self-reported cognitive performance were tested and compared to passive and active control groups. To observe cognitive performance, we used standard questions from the judgment and decision-making literature that allowed us to measure the number of intuitive (but incorrect) and reflective (and correct) answers.

A related and ongoing debate in the literature is whether any of these techniques produces socially desirable responding (cf. Protzko et al., 2019; Saribay et al., 2020). Rather than actually activating reflective or intuitive thinking, these techniques may lead participants to believe that their cognitive performance is affected. To check for this possibility, we measured self-reported beliefs about the cognitive effects of the manipulations as well as a well-established personality scale of social desirability (Hart et al., 2015).

### Review of intuition and reflection manipulations

Many techniques for activating intuitive and reflective thinking have been described in the literature. Some of these approaches, including the scrambled sentence task, the cognitive disfluency task, and visual primes such as Rodin’s *The Thinker*, have clearly failed recent replication attempts (Bakhti, 2018; Deppe et al., 2015; Meyer et al., 2015; Sanchez et al., 2017; Sirota et al., 2020; Yilmaz & Bahcekapili, 2015; Yilmaz & Saribay, 2016). We exclude those that are increasingly viewed as unreliable and focus on the following eight promising techniques.

#### Memory recall

A commonly used technique for activating intuition and reflection is the memory recall task (Cappelen et al., 2013; Forstmann & Burgmer, 2015; Ma et al., 2015; Rand et al., 2012; Shenhav et al., 2012). The technique is designed to work by recalling memories about the positive effects of relying on reflection and intuition. In a paragraph of approximately 8–10 sentences, participants in the intuitive condition are asked to describe “a time your intuition/first instinct led you in the right direction and resulted in a good outcome,” whereas participants in the reflection condition are asked to describe “a time carefully reasoning through a situation led you in the right direction and resulted in a good outcome.” Despite its widespread use, the technique failed to affect actual performance in two recent high-powered studies conducted in both WEIRD (i.e., Western, educated, industrialized, rich, and democratic; see Henrich et al., 2010) cultures (Isler et al., 2020) and non-WEIRD cultures (Saribay et al., 2020). However, these failures may reflect deviations from the original protocols, such as the lower number of sentences required in the writing task (Isler et al., 2020) or the application of the technique in a language and culture different from that of the original study (Saribay et al., 2020). Hence, we tested the effectiveness of the memory recall technique by using the original protocol among an English-speaking WEIRD sample (cf. Shenhav et al., 2012).

#### Induction prompts

A relatively new and simple technique for activating intuition and reflection is to explicitly instruct participants to rely on emotion or reason in their decisions (Gärtner et al., 2020; Kvarven et al., 2020; Levine et al., 2018; Martel et al., 2020). It is assumed that prompts to use reason will motivate reflective thinking and prompts to use emotion will motivate intuitive thinking in the decision-making tasks following these instructions. Although this technique is becoming popular in the field of cooperation (Kvarven et al., 2020), to the best of our knowledge, no previous study has tested its effects on actual cognitive performance. For this reason, we employed the standard emotion and reflection induction manipulations in our study (Levine et al., 2018).

#### Time limits

One of the most frequently used techniques to experimentally activate reflection and intuition is to impose time limits on decisions (Bouwmeester et al., 2017; Chen & Krajbich, 2018; Evans & Curtis-Holmes, 2005; Isler, Gächter, Maule, & Starmer, 2021a; Isler, Yilmaz, & Maule, 2021c; Kocher & Sutter, 2006; Maule et al., 2000; Neo et al., 2013; Payne et al., 1996; Rand, 2016; Rand et al., 2012; Sutter et al., 2003). While there is considerable variation in the use of time limits, most recent studies compare decisions made under time pressure (e.g., with prompts to decide “quickly” and “in less than 5 or 10 seconds”) to those made following a time delay (e.g., via prompts to think “carefully” and “for at least 10 or 20 seconds”). Time pressure is intended to increase reliance on intuition by cutting reflective processes short, and time delay is intended to encourage reliance on reflection by lengthening them. Despite their widespread use, time limit manipulations have various methodological drawbacks (for overviews see Horstmann et al., 2009; Spiliopoulos & Ortmann, 2014), such as task misunderstanding and noncompliance with time limits (Recalde et al., 2018; Tinghög et al., 2013). While recent studies have shown some of these limitations to be relatively harmless or have found ways of mitigating them (Goeschl & Lohse, 2018; Isler et al., 2018), the arbitrariness of the time limit durations used in the recent literature remains an important issue, and the effects of different durations on cognitive performance have not been systematically tested (Capraro & Cococcioni, 2016; Myrseth & Wollbrant, 2016; Myrseth & Wollbrant, 2017). The fact that a control group is often not used in these studies raises additional questions about whether it is time pressure or time delay that is driving the effect of the manipulations (cf. Everett et al., 2017). To shed light on these methodological issues, we implemented time limit conditions with the most commonly used durations (i.e., 5 s and 10 s time pressure and 10 s and 20 s time delay) and compared them with control conditions without time limits.

#### Cognitive load

A frequently used technique for activating intuitive thinking in the laboratory is the cognitive load manipulation. Cognitive load manipulations are designed to preoccupy the minds of the participants while they are completing other tasks that include the outcome measures, for example, by requiring them to memorize an alphanumeric string (e.g., Yilmaz & Saribay, 2016) or a dot matrix (e.g., Neys, 2006; Trémolière et al., 2012) or to continuously classify musical tones (e.g., Mieth et al., 2021). Working memory load is expected to lower the ability to use reflective thinking and to increase reliance on intuitions. Most cognitive load techniques are designed for laboratory experiments and cannot be used online due to the difficulty in checking task compliance (cf. Greene et al., 2008). For example, participants can record the piece of information given to them on their computers or smartphones (e.g., by taking a photo or a screenshot) rather than memorizing it. We designed a viable online version of the cognitive load task by requiring participants to actively use both hands to press various keyboard keys during the memorization task (see Method). Since we implemented this technique for the first time, we compared the effects of two variations involving different levels of difficulty based on the dot matrix tasks used in Trémolière et al. (2012).

#### Debiasing training

A promising technique for activating reflection is debiasing training (Isler et al., 2020), which builds on successful laboratory tests (Yilmaz & Saribay, 2017a, 2017b) and well-established debiasing principles (Lewandowsky et al., 2012; Yilmaz & Saribay, 2017a, 2017b). Although previous reflection training techniques have been shown to be effective (e.g., De Neys & Glumicic, 2008; Morewedge et al., 2015; Sellier et al., 2019; Stephens et al., 2020), their long and complex structures make it difficult to use them in online experiments. In Isler et al. (2020), we designed a brief debiasing training to increase awareness of three commonly observed cognitive biases (i.e., the semantic illusion, the base rate fallacy, and the availability bias) in the online environment and showed that it significantly improves cognitive performance on the Cognitive Reflection Test-2 (CRT-2; Thomson & Oppenheimer, 2016). In the training, participants are asked three questions demonstrating the three biases, who first answer and then receive feedback on the correct answers along with explanations of the biases. Afterward, participants are asked to summarize in writing what they have learned in training. The task ends with instructions to rely on reflection during the next task. We included in the current study the original debiasing training protocol from Isler et al. (2020) as well as a novel shortened version of it (see Method).

#### Decision justification

Another promising but neglected technique for activating reflection involves asking participants to justify their decisions. Decision justification was shown to be effective more than thirty years ago in reducing framing effects in the classic Asian disease problem (Miller & Fagley, 1991; Sieck & Yates, 1997; Takemura, 1993, 1994) as well as in increasing cognitive complexity (Tetlock & Kim, 1987) and lowering overconfidence (Arkes et al., 1987). Since then, its effectiveness was observed across multiple domains, improving health (Almashat et al., 2008) and consumption decisions (Cheng et al., 2014). Most recently, we showed in an online experiment with high statistical power that the decision justification technique, where we asked participants to justify their answers on CRT-2 by explaining their reasoning in one sentence or more, significantly increased cognitive performance (Isler et al., 2020). The technique remains underutilized in current behavior research. Therefore, we included it in the current study to retest its effectiveness and compare it with other promising techniques.

#### Monetary incentives

The use of monetary payoffs that depend on task performance is a widely accepted methodological practice in experimental economics (Smith, 1976; Voslinsky & Azar, 2021). Accordingly, monetary incentives motivate investment in cognitive effort to avoid errors of judgment, resulting in behavior that reflects underlying preferences (Hertwig & Ortmann, 2001; Vlaev, 2012). Accumulated evidence suggests that monetary incentives improve performance in particular on judgment tasks (Camerer & Hogarth, 1999); however, to the best of our knowledge, monetary incentives have not been used specifically as a technique for activating reflective thinking in the judgment-and-decision-making literature, perhaps because of the added experimental costs associated with the technique. We used an online version of the monetary incentivization technique with feasible added costs and compared it to other techniques for activating reflection.

#### Two-response elicitation

In addition to the between-subjects single-response manipulations described above, we used two versions of the within-subjects two-response elicitation technique involving time limit manipulations. Recently employed in the literature with promising results (Bago, Bonnefon, & De Neys, 2020a; Thompson et al., 2011; Yilmaz & Isler, 2019), with this technique, participants are asked to first make their decisions under time pressure and then are given an opportunity to revise their decisions with more deliberation. Hence, the technique is designed to elicit one relatively more intuitive and one relatively more reflective decision at the individual level, which often results in non-negligible magnitudes of significant effects (e.g., Bago, Rand, & Pennycook, 2020b; Boissin et al., 2021; Raoelison et al., 2020). However, it is unknown whether and how this technique activates intuition and reflection—specifically, whether the difference between the two responses is due to the time pressure in the initial response, the additional deliberation in the final one, or both manipulations. To provide insights into this question, we compared the responses elicited first under time pressure and then time delay to the active and passive control conditions without time limits. In addition to this standard two-response elicitation technique, we tested whether combining time delay with the decision justification technique described above would increase the strength of the manipulation.

### Hypotheses

We preregistered two hypotheses about the between-subjects single-response conditions and a third hypothesis for the within-subjects two-response conditions.

- H_1_: In the single-response conditions, the reflection manipulations increase cognitive reflection compared to the controls and the intuition manipulations.
- H_2_: In the single-response conditions, the intuition manipulations decrease cognitive reflection compared to the controls and the reflection manipulations.
- H_3_: In the two-response conditions, the reflection manipulations increase cognitive reflection compared to the intuition manipulations.

## Method

We obtained ethics approval from the Queensland University of Technology Human Research Ethics Committee and received informed consent from every subject prior to participation. The experiment was preregistered at the Open Science Framework (OSF; https://osf.io/rtcm5). The dataset, the experimental materials, and the analysis code are available at the OSF project site (https://osf.io/67rf4).

### Participants

Equal numbers of men and women were recruited online via Prolific (www.prolific.ac; Palan & Schitter, 2018). Recruitment was restricted to fluent English-speaking adult UK residents with Prolific approval ratings of 90 or higher, thereby excluding a few consistently noncompliant members from participation. It was announced that participation required the use of either a laptop or desktop computer, and those with any other mobile devices were not allowed to participate. As preregistered, participants with incomplete (*n* = 263) or duplicate (*n* = 7) submissions were excluded from the dataset prior to analysis. We analyze complete submissions from 3667 unique participants (*M*_age_ = 36.86, *SD*_age_ = 14.32; 50.0% female). In addition to a participation fee of £0.42, participants received £0.20 for compliance with task instructions, except for the monetary incentives condition, where participants could additionally earn up to £1.00 as detailed below.

### Planned sample size

We planned for a very powerful test (1 – *β* = 0.975) to identify small effects of manipulations (*f* = 0.10) with a standard type I error rate (*α* = 0.05) in a one-way analysis of variance (ANOVA) model of 16 conditions (i.e., excluding the two two-response conditions with within-subjects designs). Using G*Power 3.1.9.2 (Faul et al., 2009), we estimated our target sample size to include at least 3200 complete submissions or 200 participants per treatment. In addition, we aimed to recruit at least 200 participants for each of the two two-response conditions, achieving power of 0.979 to identify small effects of manipulations (*f* = 0.10) in a repeated ANOVA model with two measures and two groups. In total, we planned to recruit at least 3600 participants.

### Materials and procedure

As described in Table 1, the experiment consisted of 18 experimental conditions, including two controls, six intuition manipulations, eight reflection manipulations, and two within-subjects manipulations of first intuition and then reflection. In all but two conditions, there were two main consecutive tasks: the first task included the experimental manipulation or the active control, and the following task included the Cognitive Performance Test (CPT). The two exceptions were the passive control and the monetary incentives conditions, which involved a single main task including the CPT. Participants were randomly assigned to one of 18 conditions and remained blind to the other experimental conditions throughout the study.

**Table 1:** Study overview

Task description by experimental condition	Observations		Response time	
	Complete	Incomplete	CPT	Total
Controls				
Passive control: No manipulation or active control	202	1	93	458
Active control: Completing a neutral reading and writing task	205	3	87	584
Single-response intuition				
Intuition recall: Describing a time when intuition was beneficial	203	98	97	816
Emotion induction: Relying on emotion rather than reason	202	1	76	444
10s time pressure: Deciding within 10 seconds for each question	202	3	44	381
5s time pressure: Deciding within 5 seconds for each question	203	2	37	360
High cognitive load: Memorizing a 3x3 matrix before each question	208	12	78	568
Very high cognitive load: Memorizing a 4x4 matrix before each question	209	11	79	587
Single-response reflection				
Reason recall: Describing a time when carefully reasoning was beneficial	201	96	109	825
Reason induction: Relying on reason rather than emotion	206	1	105	497
10s time delay: Thinking carefully for at least 10 seconds for each question	202	2	100	466
20s time delay: Thinking carefully for at least 20 seconds for each question	201	3	139	500
Monetary incentives: Earning £0.20 for each correct answer	203	2	105	478
Decision justification: Providing a written explanation for each answer	209	12	389	760
Short debiasing training: Learning answers to three bias questions	205	2	113	569
Long debiasing training: Learning explanations to and writing about three bias questions	204	5	127	800
Two-response				
Standard two-response: 5s time pressure followed by 10s time delay	202	3	43, 72	492
Modified two-response: 5s time pressure followed by 10s time delay & decision justification	200	6	38, 205	625

The table describes the tasks, the number of complete and incomplete observations and median response times in seconds during the completion of the Cognitive Performance Test (CPT) and during the whole study (Total) across the experimental conditions

#### Experimental conditions

##### Passive and active controls

The only task in the passive control condition was the CPT. Hence, the passive control provides a baseline measure of cognitive performance in the participant pool. In the active control condition, participants were asked to describe an object that they own or see around them in four sentences before completing the CPT (Isler et al., 2020). The active control is intended to control for any direct effect that the writing tasks in various manipulation conditions may have on cognitive performance.

##### Intuition and reason recall

Based on Study 3 in Shenhav et al. (2012), the intuition recall and reason recall conditions included writing tasks designed to recall positive memories involving reliance on either intuition or reason. In the intuition recall, participants were asked to write a paragraph consisting of eight sentences describing an episode when their “intuition/first instinct” led them “in the right direction and resulted in a good outcome.” In contrast, participants in the reason recall condition were asked to write a paragraph consisting of eight sentences describing an episode when “carefully reasoning through a situation” led them “in the right direction and resulted in a good outcome.” In addition, each CPT question screen included either the prompt “Rely on your reason” or the prompt “Rely on your emotion.” Given previous evidence of high rates of noncompliance in these tasks, such as 21% drop-out for intuition recall in Isler et al. (2020), we recruited additional participants prior to data analysis until the target rate of 200 complete observations was reached in both conditions.

##### Emotion and reason induction

Based on Study 3 in Levine et al. (2018), this technique uses prompts to rely on either emotion or reason. The emotion induction condition stated that “Many people believe that emotion leads to good decision-making. When we use feelings, rather than logic, we make emotionally satisfying decisions.” Participants were then asked to “answer each question by relying on emotion, rather than reason.” In contrast, the reason induction condition stated that “Many people believe that reason leads to good decision-making. When we use logic, rather than feelings, we make rationally satisfying decisions.” Participants were asked to “answer each question by relying on reason, rather than emotion.” In addition, each CPT question screen included either the prompt “Remember how intuitions can help” or the prompt “Remember how reasoning can help.”

##### Time pressure and time delay

Using the standard protocol in the literature (e.g., Isler et al., 2018), the 10-second (s) time pressure condition asked participants to answer each question within 10 s. Following criticism that 10 s may not be long enough to induce intuitive decisions (Myrseth & Wollbrant, 2017), we also tested a time pressure condition with a 5 s time limit. Each CPT question screen displayed an intuition prompt (“Be quick!”) and a timer counting up from 0 s. The median response time per question and average rate of compliance with time limits across the five CPT questions were 9 s and 62.6% for the 10 s time pressure condition and 7 s and 30% for the 5 s time pressure condition (see Table 1). In contrast, participants in the time delay conditions were prompted to think carefully before answering each CPT question. Given high but preventable rates of noncompliance in previous tests of the voluntary time delay manipulation (e.g., 33% in Isler et al., 2020), we opted to use forced time delay, where participants had to wait either 10 s in the 10 s time delay condition or 20 s in the 20 s time delay condition before they could submit answers for each CPT question. The median response time per question was 20 s for 10 s time delay and 28 s for 20 s time delay. In addition, each CPT question screen displayed a reflection prompt (“Carefully consider your answer”) and a timer counting up from zero seconds. Since it was not technically possible to submit answers before the time limits, all participants with complete submissions were compliant in the time delay conditions.

##### Cognitive load

We adapted the dot memory task (e.g., Trémolière et al., 2012) to the online environment. The dot memory task works by displaying a matrix with dots in some of its cells and prompting participants to memorize the locations of these dots. To curtail cheating in the online version of the task (e.g., by taking a photo or a screenshot of the matrix), participants had to simultaneously press the “Escape” and “Backspace” keys on their keyboards to be able to see the image of the matrix. Participants had to keep pressing the keys to continue viewing the image. The image was displayed for at most 5 s or until one of the keys was no longer pressed. Because the keys are located at the opposite ends of the keyboard, this setup forces participants to use both hands simultaneously, thereby restricting opportunities for cheating. Participants saw a different dot matrix before seeing and answering each question on the CPT. Before moving to the next CPT question, participants were asked to identify the image they were shown in a multiple-choice question with four different options. As in Trémolière et al. (2012), the high cognitive load condition included 3 × 3 matrices with four dots, and the very high cognitive load condition included 4 × 4 matrices with five dots. Before the manipulation, participants received training on how to use their keyboards to view the matrices. The overall rate of compliance in the load manipulation conditions, measured as the average number of correct answers on the five multiple-choice test questions that were elicited after each question on the CPT, was 84.2% for the high cognitive load and 87.3% for the very high cognitive load conditions (see Discussion).

##### Monetary incentives

Performance on the CPT was incentivized with monetary rewards in the monetary incentives condition. Participants earned £0.20 for each correct answer, for a maximum possible reward of £1 in addition to the participation fee.

##### Decision justification

Following Isler et al. (2020), participants in the decision justification condition were given the following instructions before the CPT: “Please explain your answer to each question by writing a description of your reasoning in one sentence or more.” Each question screen included the prompt “Explain your reasoning,” and a text box for recording their explanations.

##### Debiasing training

We used two versions, the short debiasing training and the long debiasing training conditions, which were designed to improve vigilance against commonly observed cognitive biases in the online context. The long debiasing training was taken from Isler et al. (2020). In both versions, participants were asked to answer three questions testing (1) the semantic illusion, (2) the base rate fallacy, and (3) the availability bias. In the long debiasing training, participants were given feedback after each question on the correct answer together with a detailed explanation of the biases, then asked to write four sentences summarizing what they had learned in the training, and were finally instructed to rely on reflection during the CPT. In the short debiasing training, participants were given feedback on the correct answer and instructed to rely on reflection during the CPT, but no detailed explanations were provided and there was no writing task. In both versions, each CPT question screen included the prompt “Pause and reconsider your initial answer.”

##### Two-response elicitation

We used two versions of the two-response elicitation technique, a standard and a modified version (Bago, Bonnefon, & De Neys, 2020a; Thompson et al., 2011; Yilmaz & Isler, 2019). In the standard two-response condition, participants were first asked to respond to each CPT question within 5 s (i.e., as in the 5 s time pressure condition including the “Be quick!” prompt and the timer counting up from 0 s) and then asked to reflect on their initial answer to each question for at least 10 s (i.e., as in the 10 s time delay condition including the “Carefully consider your answer” prompt and the timer counting up from 0 s). In the modified two-response condition, participants were additionally asked to justify their decisions (i.e., as in the decision justification condition including the “Explain your reasoning” prompt) during the elicitation of their second responses. In both versions, the initial decision was displayed on the second decision screen (“Your initial answer was […]”) and included the prompt “Carefully consider and either revise or confirm your answer.” The median response time per question and average rate of compliance with time pressure conditions across the five consecutive CPT questions were 9 s and 23.6% for standard two-response and 8 s and 27.8% for modified two-response conditions (see Discussion). For the final answers on the CPT, the median response time per question was 14 s for the standard two-response and 41 s for the modified two-response conditions.

#### Measures

##### The cognitive performance test

As our primary outcome measure, participants in all conditions completed the CPT, which consisted of five multiple-choice questions that were randomly presented on consecutive screens (see Table 2). The CPT included the validated three-item four-option multiple-choice version (Sirota & Juanchich, 2018) of the original Cognitive Reflection Test (CRT) questions (Frederick, 2005). The options on the three CRT questions included the correct choice that reflective thinking helps to reach (i.e., the “reflective & correct” answer), an incorrect choice that tends to result from intuitive responding (the “intuitive & incorrect” answer), and two other choices that are neither intuitive nor correct (the “non-intuitive & incorrect” answers). The remaining two questions on the CPT involved well-established tasks from the heuristics-and-biases literature: a syllogistic reasoning task with belief bias (Baron et al., 2015; Markovits & Nantel, 1989) and a standard conjunction fallacy question (Kahneman & Tversky, 1973; Yilmaz & Saribay, 2017c). Both questions had multiple choices with two options: a “reflective & correct” answer and an “intuitive & incorrect” answer. Cronbach’s α for the five CPT items was .70 across all single-response conditions. As detailed in the previous section, the decision screens of experimental conditions included various prompts to improve their effectiveness, except for the active and passive control, the monetary incentives, the high cognitive load, and the very high cognitive load conditions. To facilitate the reading and comprehension of the CPT questions, the question texts became visible 2 s before the multiple choices as well as any prompts and timers that appeared on the same screen.

**Table 2:** The cognitive performance test

Question	Reflective & correct answer	Intuitive & incorrect answer	Non-intuitive & incorrect answers
1) A bat and a ball cost £1.10 in total. The bat costs £1.00 more than the ball. How much does the ball cost?	5 pence	10 pence	1 or 9 pence
2) If it takes 5 machines 5 minutes to make 5 widgets, how long would it take 100 machines to make 100 widgets?	5 minutes	100 minutes	20 or 500 minutes
3) In a lake, there is a patch of lily pads. Every day, the patch doubles in size. If it takes 48 days for the patch to cover the entire lake, how long would it take for the patch to cover half of the lake?	47 days	24 days	12 or 36 days
4) All living things need water. Roses need water. If these two statements are true, can we conclude from them that roses are living things?	No	Yes	NA
5) Claire is 31 years old, single, outspoken and very bright. She majored in philosophy. As a student, she was deeply concerned with issues of discrimination and social justice, and also participated in anti-nuclear demonstrations. Which is more probable?	Claire is a bank teller	Claire is a bank teller and is active in the feminist movement	NA

Table describes the questions and multiple choices on the Cognitive Performance Test (CPT). The first three questions, taken from the Cognitive Reflection Test, had one “reflective & correct,” one “intuitive & incorrect,” and two “non-intuitive & incorrect” answers. The last two questions, using standard belief bias and conjunction fallacy items, had one “reflective & correct” and one “intuitive & incorrect” answer

Our main cognitive performance measure is the reflection score, calculated as the number of “reflective & correct” answers on the CPT and used in our preregistered confirmatory tests. We define two additional performance measures for exploratory analysis: the intuition score, indicating reliance on intuitive thinking, calculated as the number of “intuitive & incorrect” answers on the CPT (see Cueva et al., 2016), and the error score, indicating decision errors, calculated as the number of “non-intuitive & incorrect” answers on the CPT. The reflection and the intuition scores are based on all five questions on the CPT and have a maximum possible score of 5. Since the last two questions on the CPT (i.e., the belief bias and the conjunction fallacy questions) do not have “non-intuitive & incorrect” answers, the error score is based on only the three CRT questions and has a maximum possible score of 3.

##### Self-reported reflection

Right after the completion of the CPT, participants were asked two questions on a scale ranging from 0 (“not at all”) to 10 (“very much”) about how much they relied on (1) “reason” and (2) “feelings or gut-reactions” when answering the CPT questions. The average ratings on the two questions, where the ratings on the second question were reversed, constitute the self-reported reflection score. For the two-response conditions, where participants completed the CPT twice, these self-report measures were elicited only once (i.e., after the second CPT following the reflection manipulations) to prevent any influence on CPT performance.

##### The balanced inventory of desirable responding (BIDR)

Next, the BIDR-16 scale was elicited for exploratory assessment of socially desirable responding on the actual and the self-reported reflection scores. BIDR-16 is composed of two eight-item subscales: Self-Deceptive Enhancement, which captures “honest but overly positive responding,” and Impression Management, which captures “bias towards pleasing others” (Hart et al., 2015). Cronbach’s α was .74 for Self-Deceptive Enhancement and .75 for Impression Management across all conditions.

## Results

### Confirmatory tests

#### Single-response conditions

**Fig. 1:**
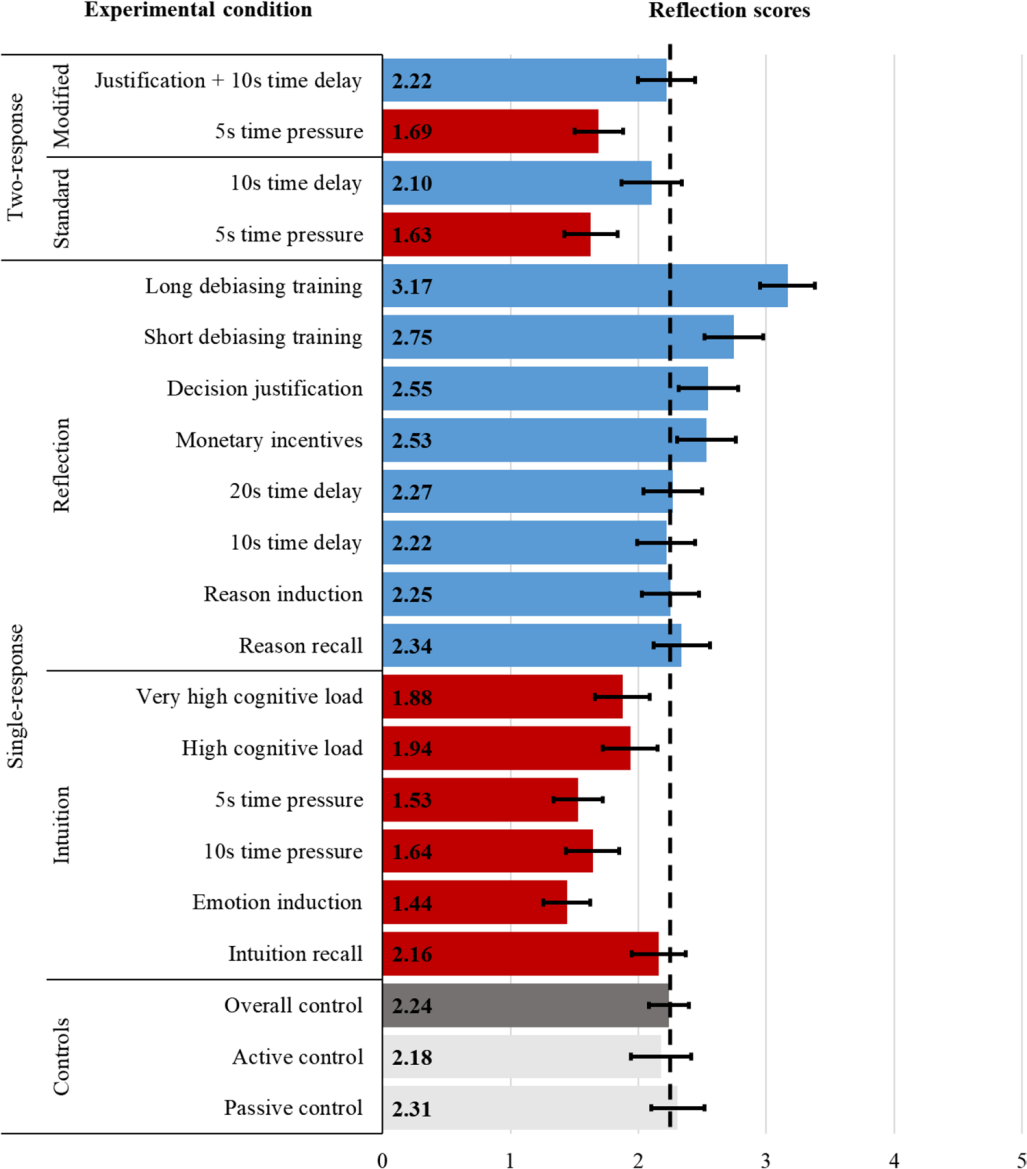
Reflection scores. The bars indicate the average reflection score (i.e., number of correct answers out of the five questions on the Cognitive Performance Test) in the control conditions (gray bars), intuition manipulations (red bars), and the reflection manipulations (blue bars) across the single-response and the two-response conditions. The dashed vertical black line indicates the average reflection score in the overall control (i.e., the pool of passive and active control conditions; *M* = 2.24, 95% CI [2.08, 2.40]). Error bars show 95% confidence intervals

Figure 1 displays the reflection scores on the CPT across all conditions. The preregistered one-way ANOVA model of the 16 single-response conditions showed significant differences in the reflection scores, *F*(15, 3249) = 16.49, *p* < .001, *η*_*p*_^*2*^ = .071. For post hoc analysis, we conducted pairwise comparisons using two-tailed independent samples *t*-tests. Failing to find a difference in the reflection scores between the active and the passive controls (*t*(405) = –0.82, *p* = .410, *d* = 0.08), we use the pool of the two control conditions (i.e., the overall control) in our confirmatory tests to increase their power and lower the number of pairwise comparisons. As summarized in Table 3, comparisons with the overall control indicated partial support for our first two hypotheses such that (H_1_) some (but not all) of the reflection manipulations increased the reflection scores and (H_2_) some (but not all) of the intuition manipulations decreased the reflection scores. The results based on the overall control are generally consistent with tests based separately on active and passive controls (see SI Table 1).

**Table 3:** Effects on cognitive reflection

	Reflection scores (%)						vs. Overall control		
	0	1	2	3	4	5	*t*	*p*	*d*
Controls									
Passive control:	14.9	19.8	15.8	24.8	18.8	5.9			
Active control:	22.4	20.0	14.6	14.6	17.1	11.2			
Overall control:	18.7	19.9	15.2	19.7	17.9	8.6			
Single-response intuition									
Intuition recall:	18.2	20.2	18.2	20.7	16.3	6.4	-0.61	.541	0.05
Emotion induction:	30.2	27.7	20.8	12.4	6.9	2.0	-6.12	< .001	0.53
10s time pressure:	28.2	27.2	16.3	12.9	10.9	4.5	-4.41	< .001	0.38
5s time pressure:	28.6	28.1	19.7	13.8	5.4	4.4	-5.39	< .001	0.46
High cognitive load:	23.6	23.1	15.9	17.3	13.9	6.3	-2.23	.026	0.19
Very high cognitive load:	25.4	22.0	16.3	18.2	12.4	5.7	-2.70	.007	0.23
Single-response reflection									
Reason recall:	17.8	14.9	17.8	21.8	19.8	7.9	0.71	.479	0.06
Reason induction:	18.0	20.4	17.5	18.0	15.1	11.2	0.08	.933	0.01
10s time delay:	20.3	16.3	21.3	15.8	15.8	10.4	-0.17	.869	0.01
20s time delay:	17.8	22.3	15.4	14.4	18.8	11.4	0.20	.843	0.02
Monetary incentives:	16.3	12.8	19.7	16.8	21.7	12.8	2.09	.037	0.18
Decision justification:	18.2	11.0	20.1	15.3	19.6	15.8	2.18	.030	0.19
Short debiasing training:	15.6	7.8	17.6	22.4	18.5	18.1	3.63	< .001	0.31
Long debiasing training:	6.9	9.3	18.6	16.2	23.5	25.5	6.79	< .001	0.58
Standard two-response									
5s time pressure:	27.1	31.0	14.8	9.9	11.8	5.4	-4.51	< .001	0.39
10s time delay:	23.2	19.2	18.2	14.3	12.3	12.8	0.97	.332	0.08
Modified two-response									
5s time pressure:	21.5	29.5	21.5	16.0	9.0	2.5	-4.18	< .001	0.36
Justification + 10s time delay:	17.5	20.5	18.5	19.0	15.0	9.5	0.15	.881	0.01

The table shows the distributions of reflection scores (%) as well as the *t*-statistics, *p*-values, and effect sizes (Cohen’s *d*s) for two-tailed independent samples *t*-tests comparing the reflection scores for each intuition and reflection manipulation across the single-response and the two-response conditions with the reflection scores in the overall control (i.e., the pool of passive and active control conditions)

Among the intuition manipulations, the emotion induction, the time pressure, and the cognitive load conditions decreased reflection as intended, but the intuition recall condition did not. Among the reflection manipulations, the debiasing training, the decision justification, and the monetary incentives conditions increased reflection as intended, but the reason recall, the reason induction, and the time delay conditions failed to do so. The cognitive load, the monetary incentives, and the decision justification conditions provided small effect sizes on reflection scores when compared against the overall control (*d* < 0.20), and consistent with this finding, these conditions significantly differed only from either the active or the passive control when compared separately.

Comparing the standard pairs of intuition-reflection manipulations frequently used together in the literature with two-tailed independent samples *t*-tests, the differences in reflection scores were significant for the induction manipulations (emotion vs. reason induction: *t*(406) = –5.51, *p* < .001, *d* = 0.55) and both pairs of time limit manipulations (10 s time pressure vs. 10 s time delay: *t*(402) = –3.68, *p* < .001, *d* = 0.37; 5 s time pressure vs. 20 s time delay: *t*(402) = –4.85, *p* < .001, *d* = 0.48), but not for the recall manipulations (intuition vs. reason recall: *t*(402) = –1.17, *p* = .243, *d* = 0.12).

#### Two-response conditions

The preregistered mixed ANOVA model showed significant overall difference in reflection scores between the first and second responses (*F*(1, 401) = 104.08, *p* < .001, *η*_*p*_^*2*^ = .206). There was no statistically significant difference between the two versions of the two-response technique (*F*(1, 401) = 0.25, *p* = .617, *η*_*p*_^*2*^ = .006) and the difference between the two responses did not depend on the version (*F*(1, 401) = 0.34, *p* = .562, η_p_^2^ < .001).

Supporting H_3_, the reflection scores were significantly lower for the initial than for the final responses in both the standard and the modified two-response conditions according to two-tailed paired samples *t*-tests (standard: *t*(201) = 7.25, *p* < .001, *d* = 0.51; modified: *t*(199) = 7.20, *p* < .001, *d* = 0.51). As detailed in Table 3, the first responses involving 5 s time pressure had lower reflection scores compared to the overall control. In contrast, the second responses involving 10 s time delay (and decision justification in the modified version) failed to go above the baseline level of reflection indicated by the overall control.

### Exploratory analyses

#### Intuition and error scores

Reflection scores were negatively correlated with both intuition (*r* = −.94, *p* < .001) and error scores (*r* = −.35, *p* < .001). While the correlation coefficient between intuition and error scores was not significant (*r* = .01, *p* = .477), one should note that this measure is by definition biased towards a negative correlation because an increase in an individual’s error score necessarily lowers the maximum possible intuition score for that individual. A tabular exploration of the data suggests that people who decide intuitively are also more likely to make non-intuitive errors. For example, the prevalence of zero error scores monotonically decreases from 88.1% to 64.3% as intuition scores increase from 0 to 4. However, this rate necessarily goes to 100% for those with intuition scores of 5, thereby hiding the positive association between the two error types.

**Fig. 2:**
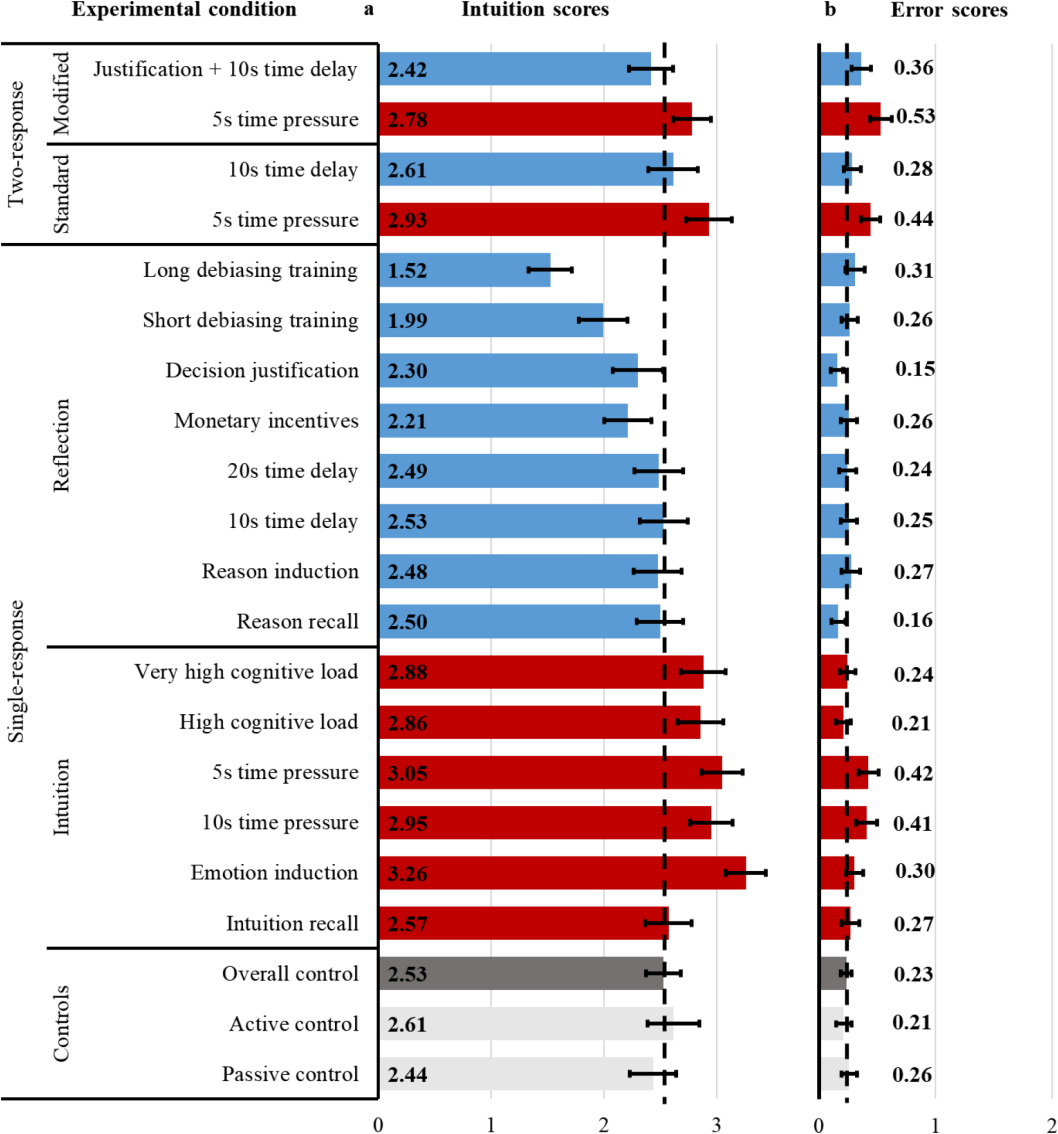
Intuition and error scores. The bars indicate the average **a **intuition score (i.e., number of intuitive & incorrect answers out of the five questions on the Cognitive Performance Test) and **b** error score (i.e., number of non-intuitive & incorrect answers out of the first three questions on the Cognitive Performance Test) in the control conditions (gray bars), the intuition manipulations (red bars), and the reflection manipulations (blue bars) across the single-response and the two-response conditions. The dashed vertical black lines indicate the average intuition score in the left panel (*M* = 2.53, 95% CI [2.37, 2.68]) and the average error score (*M* = 0.23, 95% CI [0.19, 0.28]) in the right panel for the overall control (i.e., the pool of passive and active control conditions). Error bars show 95% confidence intervals

Figure 2 displays the intuition scores on the left panel and the error scores on the right panel across the single-response and two-response conditions. The one-way ANOVA models of the single-response conditions showed significant effects on intuition scores, *F*(15, 3249) = 16.54, *p* < .001, *η*_*p*_^*2*^ *=* .071, and on error scores, *F*(15, 3249) = 3.93, *p* < .001, *η*_*p*_^*2*^ *=* .018. Table 4 reports the pairwise comparisons of intuition and error scores between each manipulation and the overall control. Except for the intuition recall condition, all single-response intuition manipulations increased intuition scores. However, the 5 s and 10 s time pressure conditions significantly increased error scores as well. The short and long debiasing training and the monetary incentives conditions decreased intuition scores. In the decision justification condition, the decrease in intuition scores failed to reach significance. On the other hand, the only condition that successfully lowered error scores was the decision justification.

**Table 4:** Effects on intuition

	Intuition scores (%)						vs. Overall control		
	*0*	*1*	*2*	*3*	*4*	*5*	*t*	*p*	*d*
Controls									
Passive control:	8.4	21.8	24.3	19.8	14.9	10.9			
Active control:	12.2	18.5	17.1	17.6	17.1	17.6			
Overall control:	10.3	20.2	20.6	18.7	16.0	14.3			
Single-response intuition									
Intuition recall:	7.4	19.7	23.7	18.2	19.7	11.3	0.35	.730	0.03
Emotion induction:	2.5	7.9	15.8	26.7	29.2	17.8	5.75	< .001	0.49
10s time pressure:	5.0	12.4	17.3	24.8	29.2	11.4	3.28	.001	0.28
5s time pressure:	5.4	5.4	20.2	30.5	24.6	13.8	4.10	< .001	0.35
High cognitive load:	6.7	14.4	19.7	20.7	22.6	15.9	2.51	.012	0.21
Very high cognitive load:	6.2	12.9	20.1	24.4	20.1	16.3	2.72	.007	0.23
Single-response reflection									
Reason recall:	8.4	20.3	24.8	19.3	14.9	12.4	-0.21	.831	0.02
Reason induction:	12.1	16.5	25.2	18.0	14.1	14.1	-0.37	.708	0.03
10s time delay:	10.9	17.3	18.3	27.7	12.9	12.9	0.03	.977	< 0.01
20s time delay:	11.4	20.3	18.8	20.3	17.3	11.9	-0.28	.776	0.02
Monetary incentives:	14.3	21.7	23.2	18.7	13.8	8.4	-2.37	.018	0.20
Decision justification:	17.2	18.7	19.1	19.6	12.4	12.9	-1.66	.097	0.14
Short debiasing training:	22.0	20.5	21.5	17.6	9.8	8.8	-3.99	< .001	0.34
Long debiasing training:	30.4	24.5	20.1	14.7	7.8	2.5	-7.74	< .001	0.66
Standard two-response									
5s time pressure:	6.9	13.3	14.3	25.6	26.6	13.3	3.09	.002	0.27
10s time delay:	13.8	14.3	14.3	25.1	20.7	11.8	0.65	.514	0.06
Modified two-response									
5s time pressure:	2.5	12.5	26.5	30.5	19.0	9.0	2.01	.045	0.17
Justification + 10s time delay:	9.5	17.5	26.0	23.5	15.5	8.0	-0.81	.419	0.07

The table shows the distributions of intuition scores (%) as well as the *t*-statistics, *p*-values, and effect sizes (Cohen’s *d*s) for two-tailed independent samples *t*-tests comparing the intuition scores for each reflection and intuition manipulation across the single-response and the two-response conditions with the intuition scores in the overall control (i.e., the pool of passive and active control conditions)

The mixed ANOVA models of the two-response conditions indicated significant differences between the initial and final responses in both the intuition scores (*F*(1, 803) = 44.94, *p* < .001, *η*_*p*_^*2*^ = .101) and the error scores (*F*(1, 803) = 27.59, *p* < .001, *η*_*p*_^*2*^ = .065). In both versions of the two-response conditions, intuition scores for the final responses were lower than the initial responses in two-tailed paired sample *t*-tests (standard: *t*(201) = –4.71, *p* < .001, *d* = 0.33; modified: *t*(199) = −4.78, *p* < .001, *d* = 0.34). Compared to the overall control, the intuition scores were higher for the initial responses but not different for the final responses (see Table 4). The initial responses, elicited under time pressure, also had higher error scores than the overall control in both two-response conditions. The final responses had lower error scores compared to the intuition manipulations according to two-tailed paired samples *t*-tests (standard: *t*(201) = –3.74, *p* < .001, *d* = 0.26; modified: *t*(199) = –3.70, *p* < .001, *d* = 0.26). However, the error scores achieved in the final responses were no different than the overall control in the standard two-response condition and remained higher than the overall control in the modified two-response condition (see Table 5).

**Table 5:** Effects on decision error

	Error scores (%)				vs. Overall control		
	*0*	*1*	*2*	*3*	*t*	*p*	*d*
Controls							
Passive control:	76.7	20.8	2.5	0.0			
Active control:	82.4	14.2	3.4	0.0			
Overall control:	79.6	17.4	3.0	0.0			
Single-response intuition							
Intuition recall:	77.8	17.7	3.9	0.5	0.85	.393	0.07
Emotion induction:	72.8	24.3	3.0	0.0	1.60	.111	0.14
10s time pressure:	66.8	26.2	6.4	0.5	3.71	< .001	0.32
5s time pressure:	64.5	28.6	6.9	0.0	4.13	< .001	0.36
High cognitive load:	81.3	16.8	1.9	0.0	0.66	.511	0.06
Very high cognitive load:	79.0	17.7	3.4	0.0	0.25	.801	0.02
Single-response reflection							
Reason recall:	86.1	11.4	2.5	0.0	-1.71	.089	0.15
Reason induction:	78.6	15.5	5.8	0.0	0.87	.383	0.07
10s time delay:	78.2	18.3	3.5	0.0	0.45	.655	0.04
20s time delay:	80.2	15.8	3.5	0.5	0.24	.811	0.02
Monetary incentives:	77.3	20.2	2.0	0.5	0.53	.594	0.05
Decision justification:	85.7	13.4	1.0	0.0	-2.07	.039	0.18
Short debiasing training:	76.6	21.0	2.0	0.5	0.71	.481	0.06
Long debiasing training:	75.0	19.6	4.9	0.5	1.68	.093	0.14
Standard two-response							
5s time pressure:	62.1	32.5	4.9	0.5	4.51	< .001	0.39
10s time delay:	75.4	21.2	3.5	0.0	1.13	.258	0.10
Modified two-response							
5s time pressure:	56.5	34.0	9.5	0.0	6.22	< .001	0.54
Justification + 10s time delay:	70.0	24.5	5.0	0.5	2.77	.006	0.24

The table shows the distributions of error scores (%) as well as the *t*-statistics, *p*-values, and effect sizes (Cohen’s *d*s) for two-tailed independent samples *t*-tests comparing the error scores for each reflection and intuition manipulation across the single-response and the two-response conditions with the error scores in the overall control (i.e., the pool of passive and active control conditions)

#### Self-reported reflection

Next, we consider the self-reported effects on reflection that are often used as manipulation checks in the literature (Isler et al., 2020; Isler, Yilmaz, & Doğruyol, 2021b; Isler, Yilmaz, & Maule, 2021c). The self-reported and actual reflection scores were positively but imperfectly correlated (*r* = .40, *p* < .001). The one-way ANOVA models including all study conditions showed significant differences in the self-reported reflection scores, *F*(17, 3649) = 64.24, *p* < .001, *η*_*p*_^*2*^ = .230. Table 6 reports the pairwise tests of the self-reported reflection scores between each treatment condition and the overall control.

**Table 6:** Effects on self-reported reflection

	vs. Overall control		
	*t*	*p*	*d*
Single-response intuition			
Intuition recall:	-5.54	< .001	0.48
Emotion induction:	-19.13	< .001	1.65
10s time pressure:	-7.23	< .001	0.62
5s time pressure:	-9.34	< .001	0.80
High cognitive load:	-0.22	.826	0.02
Very high cognitive load:	-1.28	.201	0.11
Single-response reflection			
Reason recall:	-0.52	.604	0.04
Reason induction:	10.85	< .001	0.93
10s time delay:	0.29	.772	0.02
20s time delay:	1.02	.309	0.09
Monetary incentives:	-0.28	.782	0.02
Decision justification:	2.64	.009	0.22
Short debiasing training:	0.07	.941	0.01
Long debiasing training:	4.05	< .001	0.35
Standard two-response			
	-6.39	< .001	0.55
Modified two-response			
	-6.51	< .001	0.56

The table depicts the *t*-statistics, *p*-values, and effect sizes (Cohen’s *d*s) for independent-samples *t*-tests comparing the self-reported reflection scores for each experimental condition with the overall control (i.e., the pool of passive and active control conditions)

Effects on self-reported and actual reflection scores were consistent for some of the single-response conditions (i.e., the decision justification, the long debiasing training, the emotion induction, and both time pressure manipulations). For others, there were effects on actual but not on self-reported reflection scores (i.e., the monetary incentives, the short debiasing training, and both cognitive load manipulations). For the rest, there were effects on self-reported but not on actual reflection scores (i.e., the reason induction, the intuition recall, and the two-response manipulations).

#### Socially desirable responding

The one-way ANOVA models did not show any significant differences between the study conditions in either the Self-Deceptive Enhancement, *F*(17, 3649) = 1.54, *p* = .073, *η*_*p*_^*2*^ = .007, or the Impression Management, *F*(17, 3649) = 0.47, *p* = .966, *η*_*p*_^*2*^ = .002. Although weak, both components of BIDR-16 were positively correlated with the self-reported reflection scores (Self-Deceptive Enhancement: *r* = .06, *p* < .001; Impression Management: *r* = .03, *p* = .036), and negatively correlated with the actual reflection scores (Self-Deceptive Enhancement: *r* = −.04, *p* = .011; Impression Management: *r* = −.04, *p* = .018).

## Discussion

Research on the cognitive and behavioral consequences of reflective and intuitive thinking is growing, but the usefulness of the various experimental techniques for activating intuition and reflection frequently used in the literature remain understudied. With this large-scale preregistered online experiment, we provide the first comprehensive comparison of the effects of promising reflection and intuition manipulations on cognitive performance. We identified several experimental techniques that can reliably activate reflection and intuition in the online environment. Our tests also revealed the ineffective techniques as well as those with important drawbacks.

### Effective techniques

Compared to the control benchmark, the long debiasing training was the most effective technique for increasing reflection scores and the emotion induction was the most effective technique for increasing intuition scores. As these two techniques clearly stood out from the rest, we highly recommend their use in online experiments.

Among the single-response reflection manipulations, the short debiasing training, the monetary incentives, and the decision justification techniques were also effective. These findings are consistent with those of Isler et al. (2020), who compared different reflection manipulations and found that the long debiasing training and the decision justification manipulations increase reliance on cognitive reflection.

The novel short debiasing training that we developed for online experiments is the second most powerful reflection manipulation. We recommend this technique for online studies, as it has the added advantage of taking significantly less time to implement than the long debiasing training.

The effectiveness of the monetary incentives manipulation in increasing reflection further validates the long-standing tradition of using performance payments in economic experiments (Hertwig & Ortmann, 2001). The effect achieved by this technique was relatively small, but its impact is likely to have been undermined by the fact that participants in all other conditions were also incentivized to comply with task instructions, although to smaller extents (£0.20 vs. £1.00).

We also recommend the decision justification manipulation as an alternative and promising reflection activation technique, which was shown to be effective in two recent studies and has the advantage of reducing non-intuitive decision errors (i.e., noise in data that can confound measures of intuitive decision errors). As demonstrated in Isler et al. (2020), the decision justification can be easily and successfully combined with more powerful techniques such as debiasing training.

The very high and the high cognitive load and the 5 s and the 10 s time pressure conditions were other single-response techniques effective in activating intuition. These latter findings are in line with those of Deck et al. (2017), who found significant effects for similar time pressure and cognitive load manipulations. The cognitive load manipulations validated in this study provide a novel approach to increasing task compliance in online experiments by eliminating the possibility of cheating. The Qualtrics software codes for the cognitive load (and other) techniques are publicly available at the OSF project site.

The two-response elicitation technique successfully created significant differences in cognitive performance between the initial and final responses. However, our results are consistent with previous findings (e.g., Thompson et al., 2011) that the second response elicited using this technique does not work as a reflection manipulation in absolute terms (i.e., as compared to baseline levels of reflection). Still, we recommend the two-response elicitation technique as a promising intuition manipulation, especially for studies with limited research funds, as its within-subjects design allows statistical tests with substantially higher power.

### Ineffective techniques

The remaining techniques failed. We found no evidence that the recall, the time delay, and the reason induction manipulations influenced cognitive performance as intended. Both the intuition and the reason recall were ineffective despite requiring the most resources in terms of long participant response times and high number of incomplete observations. High dropout rates, observed here as well as previously (Isler et al., 2020), are an important drawback of the recall task. High dropouts can result in differential attrition (e.g., where less patient participants are more likely to drop off in one condition more than others), which can bias comparisons with the other experimental manipulations and controls. Nevertheless, comparing the two conditions that are likely to be affected from high dropout rates and their replacement to a similar extent, namely the reason and intuition recall conditions, the task was not found to be effective. Clearly, future studies should not use the recall technique, at least as used here (e.g., in online experiments).

The time delay manipulations consistently failed to activate reflection as compared to the control benchmark, across both the 10 s and 20 s delay conditions and both the single-response and the two-response conditions. The failures of the recall and the time delay techniques are consistent with the null results reported in Isler et al. (2020). However, the time delay durations tested in our study, as in most current literature, were brief. Future studies can test the effectiveness of longer delay durations. Although it can be difficult to motivate cognitive reflection by time delay, the technique can be more effective when the decision context elicits an immediate and strong emotional response and when the time delay is long enough for its dissipation. For example, Neo et al. (2013) show that a 15-minute delay lowers the likelihood of rejection of offers in the ultimatum game, possibly due to the dissipation of initial anger. Calibration of time limits can allow for more effective pressure and delay manipulations (e.g., by estimating appropriate time limit durations based on response times that were elicited without any limits, Horstmann et al., 2009).

Similar to time delay, the reason induction manipulation also failed to increase reflection scores more than the control benchmark, suggesting that both the brief time delay manipulation frequently used in the literature and the recently popularized reason induction manipulation merely work as control groups.

Arguably, studies that have used time delay and reason-induction manipulations, such as for testing the social heuristics hypothesis (Kvarven et al., 2020; Rand, 2016) and the self-control account (Isler, Gächter, Maule, & Starmer, 2021a; Isler, Yilmaz, & Maule, 2021c), have not yet clearly considered the effects of activating reflection more than baseline levels. Previous findings relying on these manipulations should be replicated using superior techniques such as the long debiasing training, which substantially increases cognitive reflection above baseline levels, and decision justification, which lowers nonintuitive decision errors while also activating reflection.

### Drawbacks of effective techniques

Our study revealed that some of the effective techniques come with important drawbacks. In particular, there was considerable variation in effect sizes. While the effects of the long (*d =* 0.58) and the short (*d =* 0.31) debiasing training conditions were substantial, the other effective reflection manipulations involving monetary incentives (*d* = 0.18) and decision justification (*d* = 0.19) had only small-sized effects on reflection scores (but note that Isler et al., 2020, found *d* = 0.47 for decision justification compared to a passive control). Similarly, the emotion induction (*d =* 0.49) and the 5 s time pressure (*d =* 0.35) manipulations substantially increased intuition scores, but the effects of the 10 s time pressure (*d =* 0.28) and the cognitive load (*d*s ≤ 0.23) manipulations were relatively small.

Although all four time pressure conditions successfully increased intuition and decreased reflection scores, they also systematically increased non-intuitive decision errors (for a similar finding see Deck et al., 2017). The above-discussed null results of the time delay conditions suggest that it is the time pressure conditions that drive the effects of the time limit manipulations, but they do this in part by forcing participants to make mistakes and thereby introducing noise to the data. This drawback of time pressure is likely to depend on the properties of the decision task. For example, time pressure does not necessarily impair social dilemma understanding (e.g., Isler et al., 2018; Isler, Gächter, Maule, & Starmer, 2021a) or lower the quality of decisions in strategic games (Kocher & Sutter, 2006). Nevertheless, previous findings that rely on time pressure manipulations would benefit from conceptual replications using alternative intuition manipulations such as the cognitive load and the emotion induction techniques that were shown here to activate intuition without increasing non-intuitive errors.

The decision justification technique was shown to improve actual cognitive performance here as well as in previous research (Isler et al., 2020; Miller & Fagley, 1991). However, various other studies found the technique ineffective (Belardinelli et al., 2018), task-dependent (Igou & Bless, 2007; LeBoeuf & Shafir, 2003; Leisti et al., 2014), or even counterproductive (Christensen & Moynihan, 2020; Schooler et al., 1993; Sieck et al., 1999; Wilson & Schooler, 1991). Given these mixed results, further research on the underlying mechanisms, the advantages, and the potential drawbacks of the decision justification technique is needed.

### Self-reported reflection

We measured not only actual cognitive performance to examine the effects of the manipulations, but as frequently done in the literature, self-reported measures of reflection as well. For the long debiasing training, the decision justification, the emotion induction, and both time pressure manipulations, the effects on the self-reported reflection scores were significant and in the same direction as the effects on the actual reflection scores, which indicates that participants exposed to these manipulations became aware of their effects on cognitive performance. These results suggest that self-reported reflection measures can be used as manipulation checks for these techniques.

On the other hand, the self-reported reflection scores were not affected in the monetary incentives, the short debiasing training, and either of the cognitive load conditions despite significant effects on actual cognitive performance, suggesting that participants in these conditions were not aware of their effects. These techniques can be used if the research goal is to avoid creating such an awareness among participants, but self-reported manipulation checks should not be used in these cases.

In contrast, the self-reported reflection scores showed significant changes in the reason induction and the intuition recall conditions despite failures to find any impact on actual reflection scores. This indicates that participants either wrongly thought or misreported changes in cognitive performance where there was none, suggesting that relying on self-report measures as manipulation checks can be misleading for these techniques.

Finally, the self-reported reflection scores in both of the two-response conditions were lower than the overall control, even though these scores were elicited after the time delay manipulation. However, the actual reflection scores under the time delay manipulation were no different than the overall control. This is probably because the perceived or presumed effects of the initial time pressure manipulation on cognitive reflection continued during and after the time delay manipulation, whereas the actual effects of the initial time pressure manipulation were successfully eliminated with the time delay manipulation.

### Socially desirable responding

Consistent with random assignment of participants to conditions, the personality trait measures of socially desirable responding were not significantly different between the conditions. Exploratory analysis using these measures provided weak but suggestive evidence that any divergence between the self-reported and the actual cognitive performance could stem in part from desirable responding. Specifically, participants with higher actual cognitive reflection scores tended to have both lower Self-Deceptive Enhancement and Impression Management scores. In contrast, those with higher scores in these two socially desirable responding measures tended to have higher self-reported reflection scores. In other words, the discrepancy between the actual and self-reported cognitive performance increased with the tendency for socially desirable responding.

Our finding that socially desirable responding is associated with lower scores in actual cognitive reflection performance suggests an alternative explanation for the previous finding that “time pressure increases socially desirable responding” (Protzko et al., 2019). This result may indicate that time pressure has worked as intended in limiting cognitive reflection and increasing intuitive responses. It is possible that the increased reliance on intuitive thinking resulted in increased scores on measures of socially desirable responding as a byproduct of the manipulation.

Socially desirable responding is an important aspect of decision-making that needs further study. For example, asking justifications for decisions in tasks that measure prosocial intentions can motivate socially desirable responding. A similar drawback could exist for the emotion induction technique. While we have not found evidence of heightened socially desirable responding for either technique in this study, they should be tested in the context of specific applications involving moral motivations.

### Limitations and future directions

Despite providing the most comprehensive study on reflection and intuition manipulations to date, our study suffers from various limitations. Most importantly, the effectiveness of a reflection or intuition manipulation is likely to depend on the features of the task used to measure cognitive performance. For example, the effectiveness of the debiasing training could stem from the fact that both the training and the performance measurement tasks involve reasoning problems. Similarly, monetary incentives worked relatively well in our context perhaps because cognitive performance in reasoning problems with single correct answers could be objectively ranked, but this cannot be easily implemented in case of value judgments (e.g., in the context of political or religious cognition). Hence, it remains unclear whether the effectiveness of the methods identified here would generalize to other contexts and performance tasks—an important future direction for research.

Second, familiarity with the frequently used CRT questions in the CPT could have depressed the effects of the manipulations if the experienced participants in our sample were to provide answers that they had previously memorized (but see Białek & Pennycook, 2018; Meyer et al., 2018; Stagnaro et al., 2018). Although absolute levels of the effect sizes would be less informative as a result, the variation in relative effect sizes would nevertheless be insightful. Third, due to resource constraints, we did not use separate active control conditions to match the specific features of each manipulation and instead used two general control conditions. Fourth**,** to keep our survey short, we measured self-reported reflection with two simple questions rather than a validated scale with multiple items. Future studies can use scales such as Faith in Intuition (Pacini & Epstein, 1999) to more carefully observe effects on self-reported reflection. Fifth, and for the same reason of keeping our survey succinct, we did not measure the effects of these manipulations on affect. Future studies can benefit from implementing the Positive and Negative Affect Schedule (i.e., PANAS; Watson et al., 1988) as in Isler et al. (2020). Sixth, although socially desirable responding was measured using a well-established scale, its elicitation at the end of the experiment may have prevented us from observing the effects of the manipulations due to dissipation. Future methodological studies can directly test these effects on desirable responding as well as self-reported reflection without measuring actual cognitive performance beforehand. Seventh, although our experiment is high-powered, our results are restricted to participants who are Prolific members and UK residents. Hence, our findings should be replicated in more ecologically diverse settings. Finally, this study does not completely reveal the working mechanisms underlying the effective manipulations—namely, how the manipulations actually activate reflection and decrease intuition. Presumably, techniques such as the emotion induction increase intuition scores in much different ways compared to other techniques such as the cognitive load. Future studies can investigate these specific working mechanisms.

## Conclusion

This comprehensive study fills a significant methodological gap in the literature comparing intuitive and reflective decisions. Various reflection and intuition manipulation techniques tested in this study have been shown to be effective and can be easily used in future studies. In particular, we recommend the use of the debiasing training, the decision justification, and the monetary incentives techniques for activating reflection and the use of the emotion induction, the cognitive load, and the time pressure techniques for activating intuition. Some of these techniques, in particular the long debiasing training and the emotion induction, resulted in larger effect sizes than is often observed in behavioral and psychological research (Richard et al., 2003). In contrast, other techniques such as the recall, the brief time delay, and the reason induction were shown to be ineffective. The effective techniques identified here allow retesting of previous findings in the literature that were based on unreliable techniques and pave the way for novel experiments in the online context.

### Supplementary information

ESM 1 (PDF 2929 kb)
