# Supplementary material for: How to activate intuitive and reflective thinking in behavior research? A comprehensive examination of experimental techniques
Source: Behav Res Methods. 2022 Oct 17;55(7):3679–98. doi: 10.3758/s13428-022-01984-4 (PMC10615944; doi:10.3758/s13428-022-01984-4)
Supplement: Supplementary file 1 — (PDF 2929 kb) [file 13428_2022_1984_MOESM1_ESM.pdf]

Online Supplementary Information for:

How to activate intuitive and reflective thinking in behavior research? A comprehensive examination of experimental techniques

Ozan Isler[a,b*] and Onurcan Yilmaz[c]

[a] School of Economics and Finance, Queensland University of Technology, Brisbane, Australia
[b] Centre for Behavioural Economics, Society and Technology, Brisbane, Australia
[c] Department of Psychology, Kadir Has University, Istanbul, Turkey

[*] Corresponding author (ozan.isler@qut.edu.au)

Contents

Supplementary Table

SI Table 1	p.2

Experimental Instructions

Passive control	p.3
Active control	p.5
Reason recall	p.8
Intuition recall	p.12
Reason induction	p.15
Emotion induction	p.18
10s time-delay	p.21
20s time-delay	p.24
10s time-pressure	p.27
5s time-pressure	p.30
High cognitive load	p.33
Very high cognitive load	p.42
Short debiasing training	p.51
Long debiasing training	p.55
Long debiasing training	p.61
Monetary incentives	p.65
Standard two-response	p.67
Modified two-response	p.73
Self-reported reflection	p.80

SI Table 1. Effects on cognitive reflection

Manipulation vs.	Reflection Scores					
	Passive control			Active control		
	t	*p*	*d*	*t*	*p*	*d*
Single-response intuition						
Intuition recall:	-0.99	.322	0.1	-0.11	.911	0.01
Emotion induction:	-6.15	< .001	0.61	-4.84	< .001	0.48
10s time-pressure:	-4.45	< .001	0.44	-3.34	< .001	0.33
5s time-pressure:	-5.41	< .001	0.54	-4.19	< .001	0.41
High cognitive load:	-2.43	.015	0.24	-1.47	.141	0.15
Very high cognitive load:	-2.86	.005	0.28	-1.87	.063	0.18
Single-response reflection						
Reason recall:	0.21	.838	0.02	1.00	.319	0.10
Reason induction:	-0.35	.725	0.03	0.47	.641	0.05
10s time-delay:	-0.57	.568	0.06	0.25	.799	0.01
20s time-delay:	-0.24	.808	0.02	0.56	.579	0.06
Monetary incentives:	1.44	.150	0.14	2.15	.032	0.21
Decision justification:	1.50	.133	0.15	2.20	.028	0.22
Short debiasing training:	2.80	0.005	0.28	3.43	< .001	0.34
Long debiasing training:	5.67	< .001	0.56	6.13	< .001	0.61
Standard two-response						
5s time-pressure:	-4.53	< .001	0.45	-3.42	< .001	0.34
10s time-delay:	-1.27	.203	0.13	-0.42	0.672	0.04
Modified two-response						
5s time-pressure:	-4.34	< .001	0.43	-3.17	.002	0.32
Justification + 10s time-delay:	-0.57	.572	0.06	0.27	0.787	0.03

Note. The table depicts the *t*-statistics, *p*-values, and effect sizes (Cohen's *d*s) for two-tailed independent samples *t*-tests comparing the reflection scores for each intuition and reflection manipulation across the single-response and the two-response conditions with the reflection scores separately in the passive and active control conditions.

Experimental Instructions

Headings and descriptions (not seen participants) are noted in blue. Shown are the screenshots as seen by the participants. Each header denotes a separate screen.

Passive control

Overview

Overview

There is only one task in this study: Task A.

You will earn £0.20 in addition to the participation fee of £0.42 if you closely follow all of the underlined instructions in this task.

Please continue to start Task A.

Task A

Task A

For this task, we ask you to answer five test questions in a row.

Your goal is to give as many correct answers as possible.

Note: CPT questions and answers were randomly presented, and the question text became visible two seconds before the answers.

CPT Q1

A bat and a ball cost £1.10 in total. The bat costs £1.00 more than the ball. How much does the ball cost?

- ○ 10 pence
- ○ 9 pence
- ○ 1 pence
- ○ 5 pence

CPT Q2

If it takes 5 machines 5 minutes to make 5 widgets, how long would it take 100 machines to make 100 widgets?

- ◯ 500 minutes
- ◯ 100 minutes
- ◯ 20 minutes
- ◯ 5 minutes

CPT Q3

In a lake, there is a patch of lily pads. Every day, the patch doubles in size. If it takes 48 days for the patch to cover the entire lake, how long would it take for the patch to cover half of the lake?

- ◯ 24 days
- ◯ 12 days
- ◯ 36 days
- ◯ 47 days

CPT Q4

- All living things need water.
- Roses need water.

If these two statements are true, can we conclude from them that roses are living things?

- ◯ Yes
- ◯ No

CPT Q5

Claire is 31 years old, single, outspoken and very bright. She majored in philosophy. As a student, she was deeply concerned with issues of discrimination and social justice, and also participated in anti-nuclear demonstrations.

Which is more probable?

- ◯ Claire is a bank teller
- ◯ Claire is a bank teller and is active in the feminist movement

Active control

Overview

Overview

There are two tasks in this study: Task A and Task B.

You will earn £0.20 in addition to the participation fee of £0.42 if you closely follow all of the underlined instructions in both tasks.

Please continue to start Task A.

Task A

Task A

Please think of an object that you own or choose an object that you see around you. Describe this object by constructing four full sentences (one sentence in each of the four cells below).

For example: "There is a cup on my desk. The cup is filled with water. This is my favorite cup. My best friend gave it to me as a gift."

1)

2)

3)

4)

Task B

Task B

For this task, we ask you to answer five test questions in a row.

Your goal is to give as many correct answers as possible.

Note: CPT questions and answers were randomly presented, and the question text became visible two seconds before the answers.

CPT Q1

A bat and a ball cost £1.10 in total. The bat costs £1.00 more than the ball. How much does the ball cost?

- ○ 10 pence
- ○ 9 pence
- ○ 1 pence
- ○ 5 pence

CPT Q2

If it takes 5 machines 5 minutes to make 5 widgets, how long would it take 100 machines to make 100 widgets?

- ○ 500 minutes
- ○ 100 minutes
- ○ 20 minutes
- ○ 5 minutes

CPT Q3

In a lake, there is a patch of lily pads. Every day, the patch doubles in size. If it takes 48 days for the patch to cover the entire lake, how long would it take for the patch to cover half of the lake?

◯ 24 days
◯ 12 days
◯ 36 days
◯ 47 days

CPT Q4

- All living things need water.
- Roses need water.

If these two statements are true, can we conclude from them that roses are living things?

◯ Yes
◯ No

CPT Q5

Claire is 31 years old, single, outspoken and very bright. She majored in philosophy. As a student, she was deeply concerned with issues of discrimination and social justice, and also participated in anti-nuclear demonstrations.

Which is more probable?

◯ Claire is a bank teller
◯ Claire is a bank teller and is active in the feminist movement

Reason recall

Overview

Overview

There are two tasks in this study: Task A and Task B.

You will earn £0.20 in addition to the participation fee of £0.42 <u>if you closely follow all of the underlined instructions in both tasks.</u>

Please continue to start Task A.

Task A

Task A

<u>Please write a paragraph consisting of eight sentences (one sentence in each of the eight cells below)</u> describing a time carefully reasoning through a situation led you in the right direction and resulted in a good outcome.

1)

2)

3)

4)

5)

6)

[ ]

7)

[ ]

8)

[ ]

Task B

Task B

For this task, we ask you to answer five test questions in a row.

Your goal is to give as many correct answers as possible.

Note: CPT questions and answers were randomly presented, and the question text became visible two seconds before the answers.

CPT Q1

A bat and a ball cost £1.10 in total. The bat costs £1.00 more than the ball. How much does the ball cost?

Remember how reasoning can help.

- ◯ 10 pence
- ◯ 9 pence
- ◯ 1 pence
- ◯ 5 pence

CPT Q2

If it takes 5 machines 5 minutes to make 5 widgets, how long would it take 100 machines to make 100 widgets?

Remember how reasoning can help.

- ◯ 500 minutes
- ◯ 100 minutes
- ◯ 20 minutes
- ◯ 5 minutes

CPT Q3

In a lake, there is a patch of lily pads. Every day, the patch doubles in size. If it takes 48 days for the patch to cover the entire lake, how long would it take for the patch to cover half of the lake?

Remember how reasoning can help.

- ◯ 24 days
- ◯ 12 days
- ◯ 36 days
- ◯ 47 days

CPT Q4

- All living things need water.
- Roses need water.

If these two statements are true, can we conclude from them that roses are living things?

Remember how reasoning can help.

- ◯ Yes
- ◯ No

CPT Q5

Claire is 31 years old, single, outspoken and very bright. She majored in philosophy. As a student, she was deeply concerned with issues of discrimination and social justice, and also participated in anti-nuclear demonstrations.

Which is more probable?

Remember how reasoning can help.

- ○ Claire is a bank teller
- ○ Claire is a bank teller and is active in the feminist movement

Intuition recall

Overview

Overview

There are two tasks in this study: Task A and Task B.

You will earn £0.20 in addition to the participation fee of £0.42 if you closely follow all of the underlined instructions in both tasks.

Please continue to start Task A.

Task A

Task A

Please write a paragraph consisting of eight sentences (one sentence in each of the eight cells below) describing a time your intuition/first instinct led you in the right direction and resulted in a good outcome.

1)

2)

3)

4)

5)

6)

[ ]

7)

[ ]

8)

[ ]

Task B

Task B

For this task, we ask you to answer five test questions in a row.

Your goal is to give as many correct answers as possible.

Note: CPT questions and answers were randomly presented, and the question text became visible two seconds before the answers.

CPT Q1

A bat and a ball cost £1.10 in total. The bat costs £1.00 more than the ball. How much does the ball cost?

Remember how intuitions can help.

- ○ 10 pence
- ○ 9 pence
- ○ 1 pence
- ○ 5 pence

CPT Q2

If it takes 5 machines 5 minutes to make 5 widgets, how long would it take 100 machines to make 100 widgets?

Remember how intuitions can help.

- ◯ 500 minutes
- ◯ 100 minutes
- ◯ 20 minutes
- ◯ 5 minutes

CPT Q3

In a lake, there is a patch of lily pads. Every day, the patch doubles in size. If it takes 48 days for the patch to cover the entire lake, how long would it take for the patch to cover half of the lake?

Remember how intuitions can help.

- ◯ 24 days
- ◯ 12 days
- ◯ 36 days
- ◯ 47 days

CPT Q4

- All living things need water.
- Roses need water.

If these two statements are true, can we conclude from them that roses are living things?

Remember how intuitions can help.

- ◯ Yes
- ◯ No

CPT Q5

Claire is 31 years old, single, outspoken and very bright. She majored in philosophy. As a student, she was deeply concerned with issues of discrimination and social justice, and also participated in anti-nuclear demonstrations.

Which is more probable?

Remember how intuitions can help.

- ◯ Claire is a bank teller
- ◯ Claire is a bank teller and is active in the feminist movement

Reason induction

Overview

Overview

There is only one task in this study: Task A.

You will earn £0.20 in addition to the participation fee of £0.42 if you closely follow all of the underlined instructions in this task.

Please continue to start Task A.

Task A

Task A

For this task, we ask you to answer five test questions in a row.

Your goal is to give as many correct answers as possible.

Task A continued

Task A

Sometimes people make decisions by using logic and relying on their reason. Other times, people make decisions by using feeling and relying on their emotion.

Many people believe that reason leads to good decisionmaking. When we use logic, rather than feelings, we make rationally satisfying decisions.

Please answer each question by relying on reason, rather than emotion.

Task B

Task B

For this task, we ask you to <u>answer five test questions in a row.</u>

Your goal is to give as many correct answers as possible.

Note: CPT questions and answers were randomly presented, and the question text became visible two seconds before the answers.

CPT Q1

A bat and a ball cost £1.10 in total. The bat costs £1.00 more than the ball. How much does the ball cost?

Rely on your reason.

- ○ 10 pence
- ○ 9 pence
- ○ 1 pence
- ○ 5 pence

CPT Q2

If it takes 5 machines 5 minutes to make 5 widgets, how long would it take 100 machines to make 100 widgets?

Rely on your reason.

- ○ 500 minutes
- ○ 100 minutes
- ○ 20 minutes
- ○ 5 minutes

CPT Q3

In a lake, there is a patch of lily pads. Every day, the patch doubles in size. If it takes 48 days for the patch to cover the entire lake, how long would it take for the patch to cover half of the lake?

Rely on your reason.

- ◯ 24 days
- ◯ 12 days
- ◯ 36 days
- ◯ 47 days

CPT Q4

- All living things need water.
- Roses need water.

If these two statements are true, can we conclude from them that roses are living things?

Rely on your reason.

- ◯ Yes
- ◯ No

CPT Q5

Claire is 31 years old, single, outspoken and very bright. She majored in philosophy. As a student, she was deeply concerned with issues of discrimination and social justice, and also participated in anti-nuclear demonstrations.

Which is more probable?

Rely on your reason.

- ◯ Claire is a bank teller
- ◯ Claire is a bank teller and is active in the feminist movement

Emotion induction

Overview

Overview

There is only one task in this study: Task A.

You will earn £0.20 in addition to the participation fee of £0.42 if you closely follow all of the underlined instructions in this task.

Please continue to start Task A.

Task A

Task A

For this task, we ask you to answer five test questions in a row.

Your goal is to give as many correct answers as possible.

Task A continued

Task A

Sometimes people make decisions by using feeling and relying on their emotion. Other times, people make decisions by using logic and relying on their reason.

Many people believe that emotion leads to good decisionmaking. When we use feelings, rather than logic, we make emotionally satisfying decisions.

Please answer each question by relying on emotion, rather than reason.

Task B

Task B

For this task, we ask you to answer five test questions in a row.

Your goal is to give as many correct answers as possible.

Note: CPT questions and answers were randomly presented, and the question text became visible two seconds before the answers.

CPT Q1

A bat and a ball cost £1.10 in total. The bat costs £1.00 more than the ball. How much does the ball cost?

Rely on your emotion.

- ○ 10 pence
- ○ 9 pence
- ○ 1 pence
- ○ 5 pence

CPT Q2

If it takes 5 machines 5 minutes to make 5 widgets, how long would it take 100 machines to make 100 widgets?

Rely on your emotion.

- ○ 500 minutes
- ○ 100 minutes
- ○ 20 minutes
- ○ 5 minutes

CPT Q3

In a lake, there is a patch of lily pads. Every day, the patch doubles in size. If it takes 48 days for the patch to cover the entire lake, how long would it take for the patch to cover half of the lake?

Rely on your emotion.

- ○ 24 days
- ○ 12 days
- ○ 36 days
- ○ 47 days

CPT Q4

- All living things need water.
- Roses need water.

If these two statements are true, can we conclude from them that roses are living things?

Rely on your emotion.

◯ Yes
◯ No

CPT Q5

Claire is 31 years old, single, outspoken and very bright. She majored in philosophy. As a student, she was deeply concerned with issues of discrimination and social justice, and also participated in anti-nuclear demonstrations.

Which is more probable?

Rely on your emotion.

◯ Claire is a bank teller
◯ Claire is a bank teller and is active in the feminist movement

10s time-delay

Overview

Overview

There is only one task in this study: Task A.

You will earn £0.20 in addition to the participation fee of £0.42 if you closely follow all of the underlined instructions in this task.

Please continue to start Task A.

Task A

Task A

For this task, we ask you to answer five test questions in a row.

Your goal is to give as many correct answers as possible.

Task A continued

Task A

We ask that you think for at least 10 seconds before answering each question.

Please answer each question after carefully considering your decision.

On each question, the "Continue" button will appear after 10 seconds.

Note: CPT questions and answers were randomly presented, the question text and the counter (started counting and) became visible two seconds before the answers and the "Continue" button appeared 10 seconds after the answers became visible.

CPT Q1

A bat and a ball cost £1.10 in total. The bat costs £1.00 more than the ball. How much does the ball cost?

Carefully consider your answer.

- ◯ 10 pence
- ◯ 9 pence
- ◯ 1 pence
- ◯ 5 pence

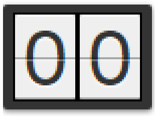

CPT Q2

If it takes 5 machines 5 minutes to make 5 widgets, how long would it take 100 machines to make 100 widgets?

Carefully consider your answer.

- ◯ 500 minutes
- ◯ 100 minutes
- ◯ 20 minutes
- ◯ 5 minutes

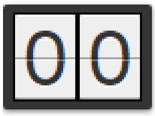

CPT Q3

In a lake, there is a patch of lily pads. Every day, the patch doubles in size. If it takes 48 days for the patch to cover the entire lake, how long would it take for the patch to cover half of the lake?

Carefully consider your answer.

- ◯ 24 days
- ◯ 12 days
- ◯ 36 days
- ◯ 47 days

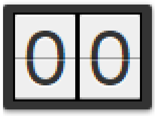

CPT Q4

- All living things need water.
- Roses need water.

If these two statements are true, can we conclude from them that roses are living things?

Carefully consider your answer.

◯ Yes
◯ No

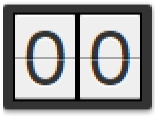

CPT Q5

Claire is 31 years old, single, outspoken and very bright. She majored in philosophy. As a student, she was deeply concerned with issues of discrimination and social justice, and also participated in anti-nuclear demonstrations.

Which is more probable?

Carefully consider your answer.

◯ Claire is a bank teller
◯ Claire is a bank teller and is active in the feminist movement

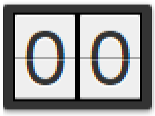

20s time-delay

Overview

Overview

There is only one task in this study: Task A.

You will earn £0.20 in addition to the participation fee of £0.42 if you closely follow all of the underlined instructions in this task.

Please continue to start Task A.

Task A

Task A

For this task, we ask you to answer five test questions in a row.

Your goal is to give as many correct answers as possible.

Task A continued

Task A

We ask that you think for at least 20 seconds before answering each question.

Please answer each question after carefully considering your decision.

On each question, the "Continue" button will appear after 20 seconds.

Note: CPT questions and answers were randomly presented, the question text and the counter (started counting and) became visible two seconds before the answers and the "Continue" button appeared 20 seconds after the answers became visible.

CPT Q1

A bat and a ball cost £1.10 in total. The bat costs £1.00 more than the ball. How much does the ball cost?

Carefully consider your answer.

- 10 pence
- 9 pence
- 1 pence
- 5 pence

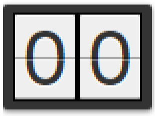

CPT Q2

If it takes 5 machines 5 minutes to make 5 widgets, how long would it take 100 machines to make 100 widgets?

Carefully consider your answer.

- 500 minutes
- 100 minutes
- 20 minutes
- 5 minutes

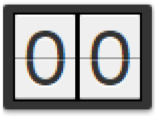

CPT Q3

In a lake, there is a patch of lily pads. Every day, the patch doubles in size. If it takes 48 days for the patch to cover the entire lake, how long would it take for the patch to cover half of the lake?

Carefully consider your answer.

- 24 days
- 12 days
- 36 days
- 47 days

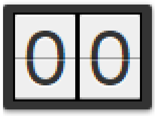

CPT Q4

- All living things need water.
- Roses need water.

If these two statements are true, can we conclude from them that roses are living things?

Carefully consider your answer.

○ Yes
○ No

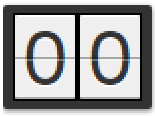

CPT Q5

Claire is 31 years old, single, outspoken and very bright. She majored in philosophy. As a student, she was deeply concerned with issues of discrimination and social justice, and also participated in anti-nuclear demonstrations.

Which is more probable?

Carefully consider your answer.

○ Claire is a bank teller
○ Claire is a bank teller and is active in the feminist movement

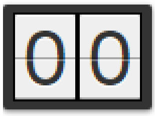

10s time-pressure

Overview

Overview

There is only one task in this study: Task A.

You will earn £0.20 in addition to the participation fee of £0.42 if you closely follow all of the underlined instructions in this task.

Please continue to start Task A.

Task A

Task A

For this task, we ask you to answer five test questions in a row.

Your goal is to give as many correct answers as possible.

Task A continued

Task A

We ask that you answer each question within 10 seconds.

Please answer each question by quickly indicating your immediate response.

Note: CPT questions and answers were randomly presented, the question text and the counter (started counting and) became visible two seconds before the answers.

CPT Q1

A bat and a ball cost £1.10 in total. The bat costs £1.00 more than the ball. How much does the ball cost?

Be quick!

○ 10 pence
○ 9 pence
○ 1 pence
○ 5 pence

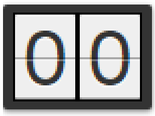

CPT Q2

If it takes 5 machines 5 minutes to make 5 widgets, how long would it take 100 machines to make 100 widgets?

Be quick!

○ 500 minutes
○ 100 minutes
○ 20 minutes
○ 5 minutes

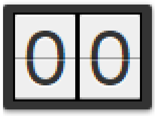

CPT Q3

In a lake, there is a patch of lily pads. Every day, the patch doubles in size. If it takes 48 days for the patch to cover the entire lake, how long would it take for the patch to cover half of the lake?

Be quick!

○ 24 days
○ 12 days
○ 36 days
○ 47 days

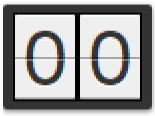

CPT Q4

- All living things need water.
- Roses need water.

If these two statements are true, can we conclude from them that roses are living things?

Be quick!

○ Yes
○ No

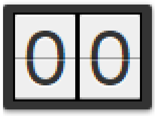

CPT Q5

Claire is 31 years old, single, outspoken and very bright. She majored in philosophy. As a student, she was deeply concerned with issues of discrimination and social justice, and also participated in anti-nuclear demonstrations.

Which is more probable?

Be quick!

○ Claire is a bank teller
○ Claire is a bank teller and is active in the feminist movement

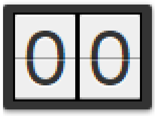

5s time-pressure

Overview

Overview

There is only one task in this study: Task A.

You will earn £0.20 in addition to the participation fee of £0.42 if you closely follow all of the underlined instructions in this task.

Please continue to start Task A.

Task A

Task A

For this task, we ask you to answer five test questions in a row.

Your goal is to give as many correct answers as possible.

Task A continued

Task A

We ask that you answer each question within 5 seconds.

Please answer each question by quickly indicating your immediate response.

Note: CPT questions and answers were randomly presented, the question text and the counter (started counting and) became visible two seconds before the answers.

CPT Q1

A bat and a ball cost £1.10 in total. The bat costs £1.00 more than the ball. How much does the ball cost?

Be quick!

- ◯ 10 pence
- ◯ 9 pence
- ◯ 1 pence
- ◯ 5 pence

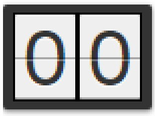

CPT Q2

If it takes 5 machines 5 minutes to make 5 widgets, how long would it take 100 machines to make 100 widgets?

Be quick!

- ◯ 500 minutes
- ◯ 100 minutes
- ◯ 20 minutes
- ◯ 5 minutes

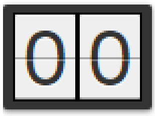

CPT Q3

In a lake, there is a patch of lily pads. Every day, the patch doubles in size. If it takes 48 days for the patch to cover the entire lake, how long would it take for the patch to cover half of the lake?

Be quick!

- ◯ 24 days
- ◯ 12 days
- ◯ 36 days
- ◯ 47 days

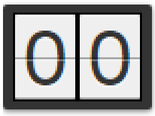

CPT Q4

- All living things need water.
- Roses need water.

If these two statements are true, can we conclude from them that roses are living things?

Be quick!

◯ Yes
◯ No

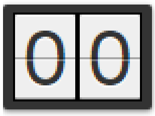

CPT Q5

Claire is 31 years old, single, outspoken and very bright. She majored in philosophy. As a student, she was deeply concerned with issues of discrimination and social justice, and also participated in anti-nuclear demonstrations.

Which is more probable?

Be quick!

◯ Claire is a bank teller
◯ Claire is a bank teller and is active in the feminist movement

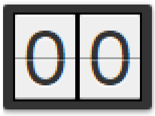

High cognitive load

Overview

Overview

There is only one task in this study: Task A.

You will earn £0.20 in addition to the participation fee of £0.42 if you closely follow all of the underlined instructions in this task.

Please continue to start Task A.

Task A

Task A

For this task, we ask you to answer five test questions in a row.

Your goal is to give as many correct answers as possible.

Task A continued

Task A

We ask that you memorize an image before answering each question.

The following screens will describe the memorization task in more detail.

Task A continued

An example of a matrix image is shown below.

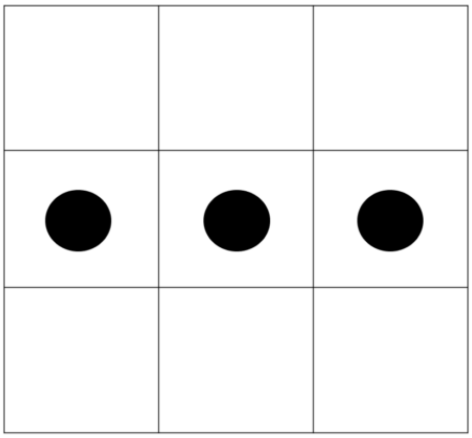

Task A continued

To be able to see the image, you will need to simultaneously press and keep pressing the "Escape" and "Backspace" keys on your keyboard. The position of these keys are shown below in red circles. You should use your left hand to press "Escape" and your right hand to press "Backspace".

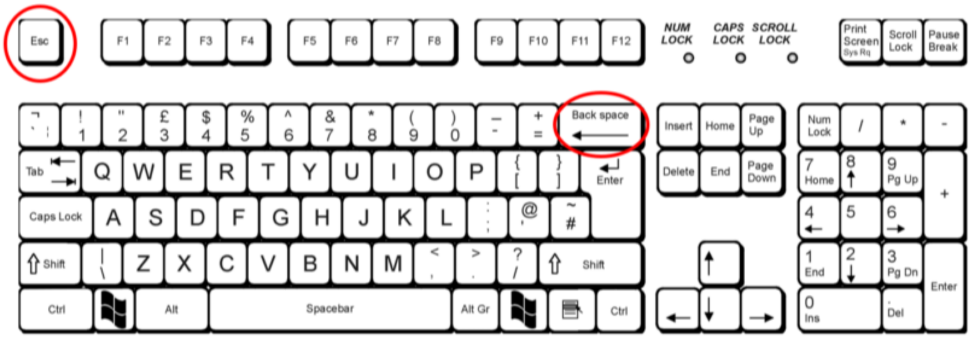

The image will be displayed for 5 seconds or until you stop pushing one or both buttons, whichever comes earlier.

You can practice viewing the image on the next screen.

Task A continued

This is a practice screen. To view the image, please push "Escape" and "Backspace" buttons at the same time. Keep pushing the two buttons until the next screen appears or until you memorize the position of the dots.

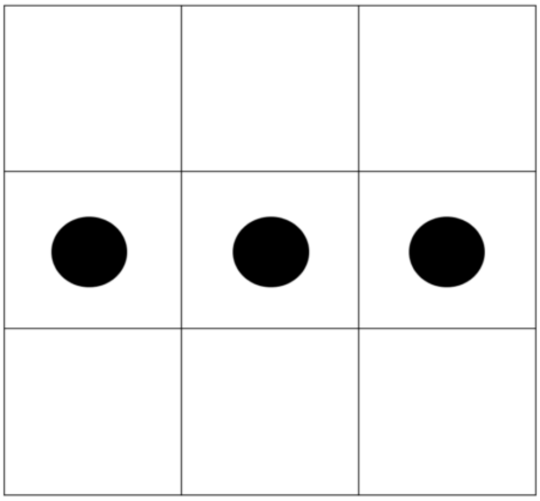

Task A continued

You need to memorize the displayed image before answering each question and keep this image in your mind while answering the question.

You will be asked to correctly identify the image after having answered the question.

You can click "Back" to practice viewing the image or click "Continue" to start Task A.

Note: CPT questions and answers were randomly presented, and the question text became visible two seconds before the answers.

CPT Q1 – Load

Please view the image by pushing "Escape" and "Backspace" buttons at the same time. You have at most 5 seconds to memorize the figure.

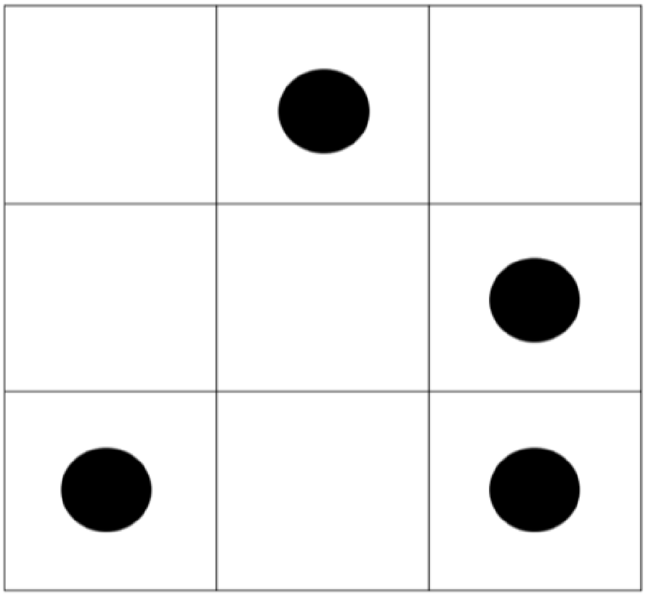

CPT Q1 - Question

A bat and a ball cost £1.10 in total. The bat costs £1.00 more than the ball. How much does the ball cost?

- ○ 10 pence
- ○ 9 pence
- ○ 1 pence
- ○ 5 pence

CPT Q1 – Load test

Please choose the image that you saw right before answering the previous question.

○ 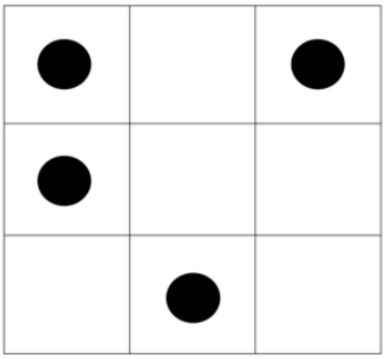

○ 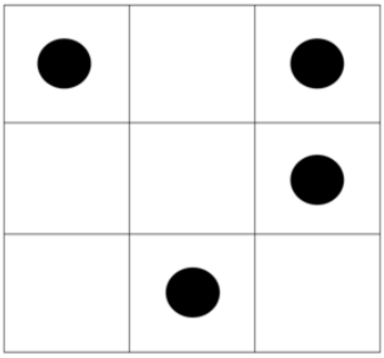

○ 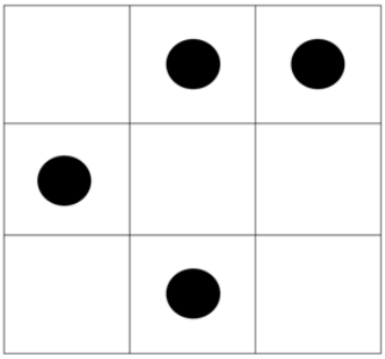

○ 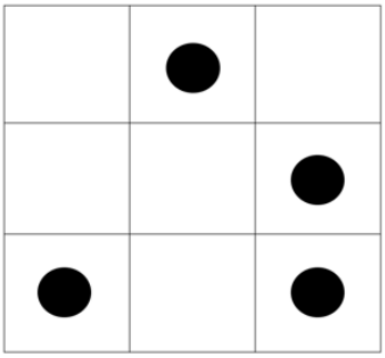

CPT Q2 – Load

Please view the image by pushing "Escape" and "Backspace" buttons at the same time.
You have at most 5 seconds to memorize the figure.

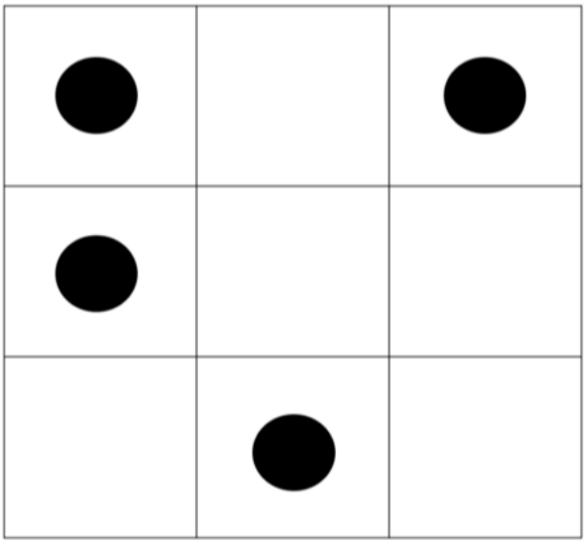

CPT Q2 – Question

If it takes 5 machines 5 minutes to make 5 widgets, how long would it take 100 machines to make 100 widgets?

○ 500 minutes
○ 100 minutes
○ 20 minutes
○ 5 minutes

CPT Q2 – Load test

Please choose the image that you saw right before answering the previous question.

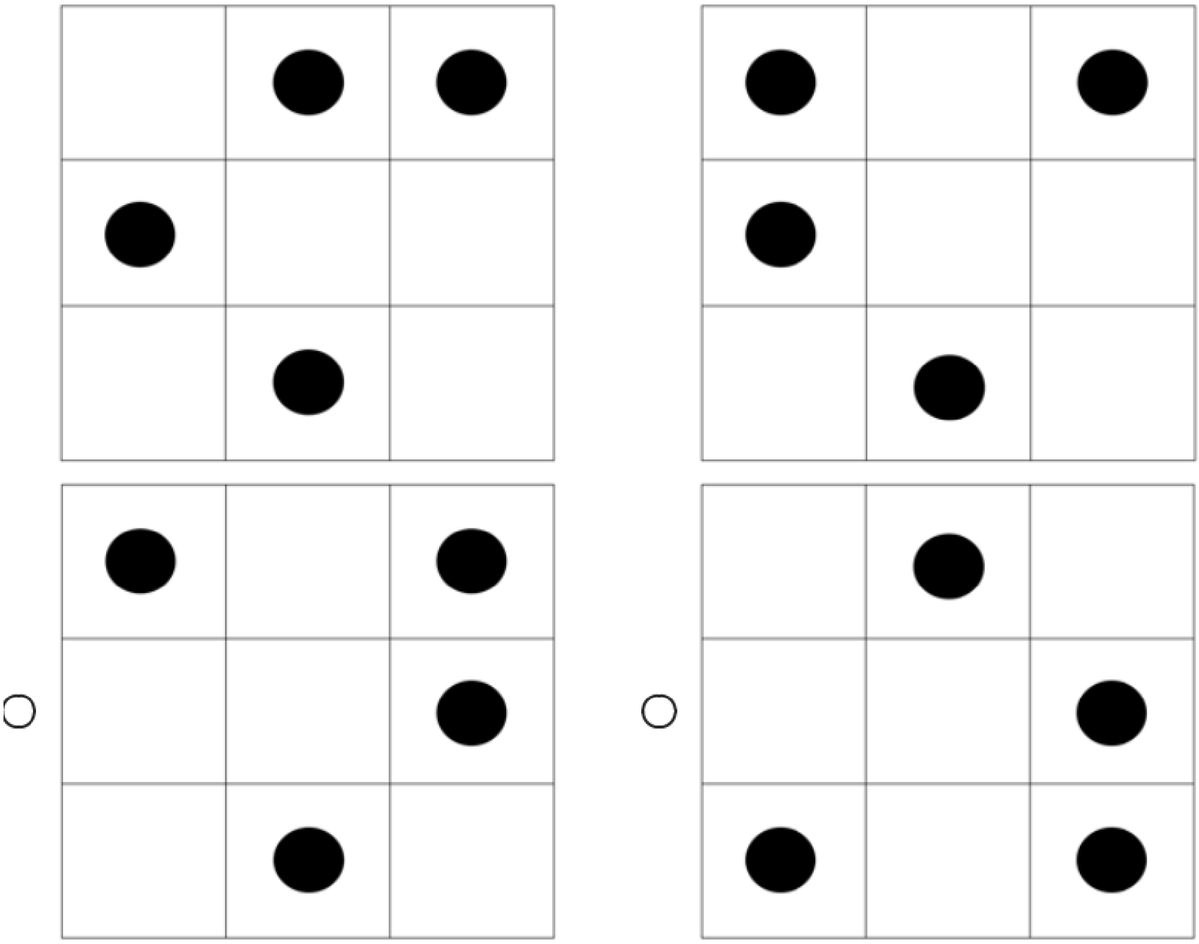

CPT Q3 – Load

Please view the image by pushing "Escape" and "Backspace" buttons at the same time. You have at most 5 seconds to memorize the figure.

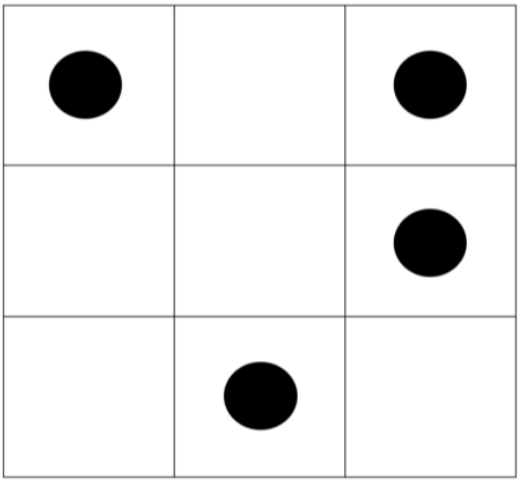

CPT Q3 – Question

In a lake, there is a patch of lily pads. Every day, the patch doubles in size. If it takes 48 days for the patch to cover the entire lake, how long would it take for the patch to cover half of the lake?

- ◯ 24 days
- ◯ 12 days
- ◯ 36 days
- ◯ 47 days

CPT Q3 – Load test

Please choose the image that you saw right before answering the previous question.

◯ 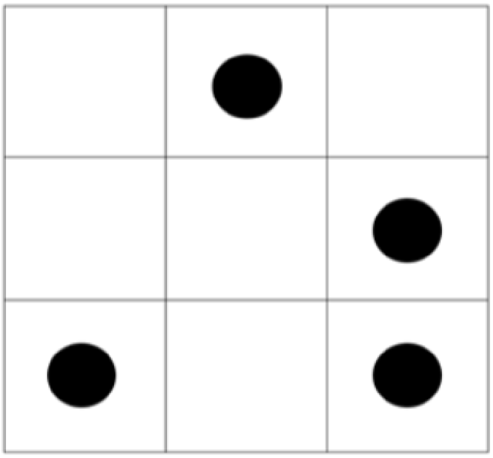

◯ 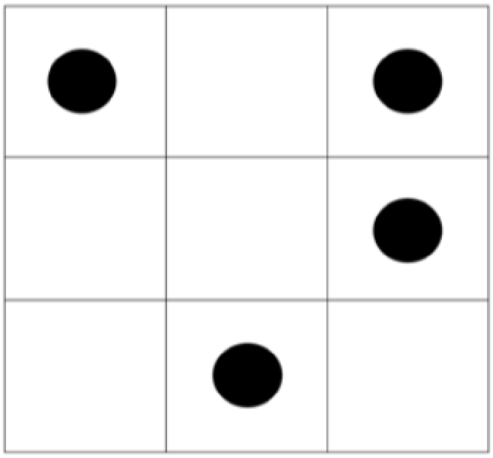

○ 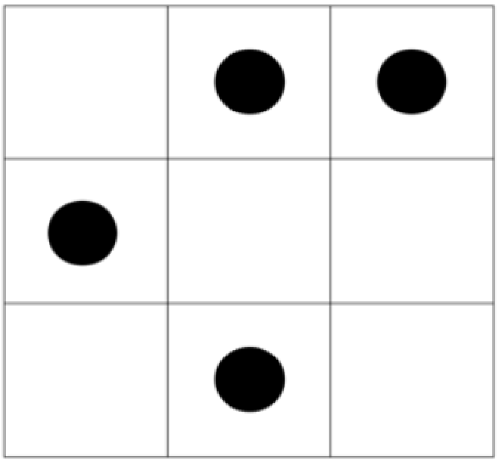 ○ 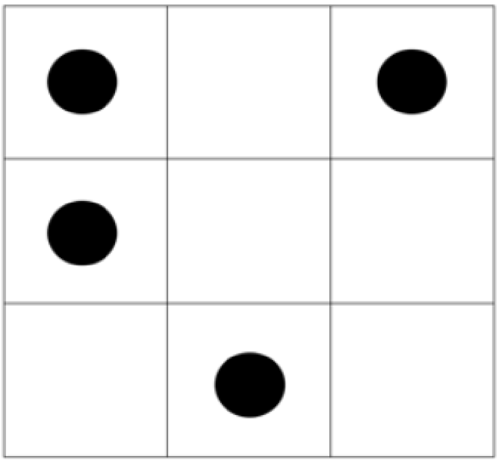

CPT Q4 – Load

Please view the image by pushing "Escape" and "Backspace" buttons at the same time. You have at most 5 seconds to memorize the figure.

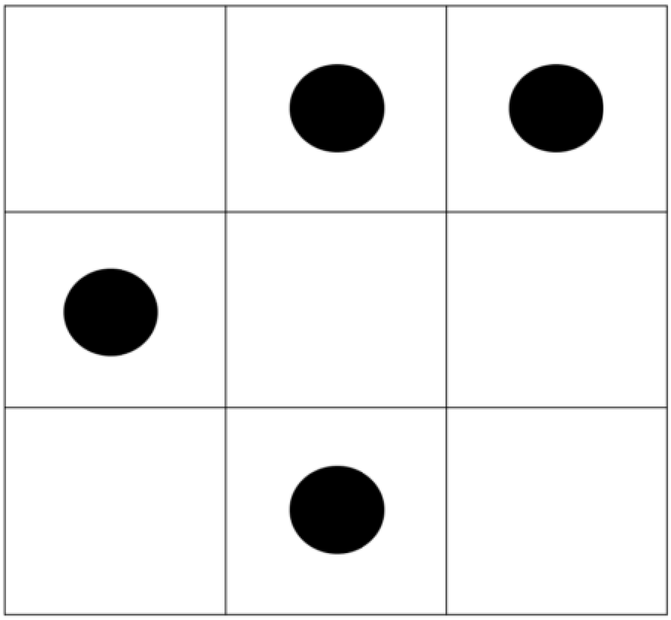

CPT Q4 – Question

- All living things need water.
- Roses need water.

If these two statements are true, can we conclude from them that roses are living things?

○ Yes
○ No

CPT Q4 – Load test

Please choose the image that you saw right before answering the previous question.

○ 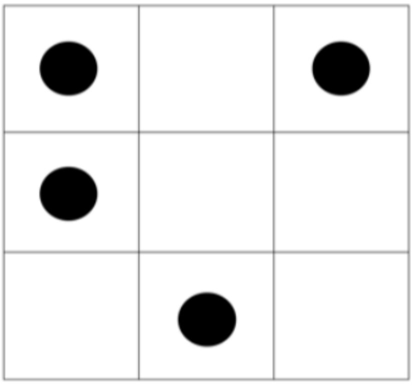

○ 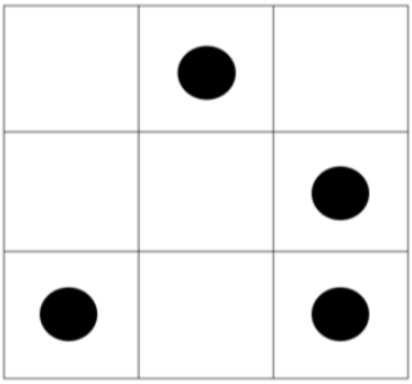

○ 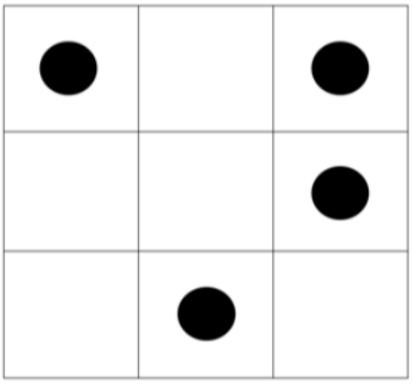

○ 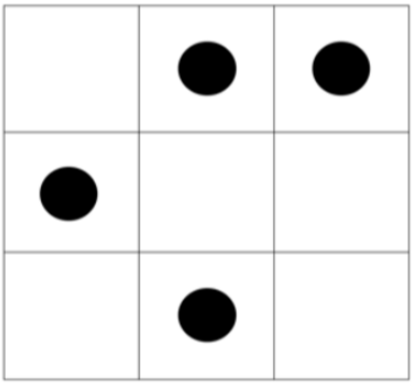

CPT Q5 – Load

Please view the image by pushing "Escape" and "Backspace" buttons at the same time.
You have at most 5 seconds to memorize the figure.

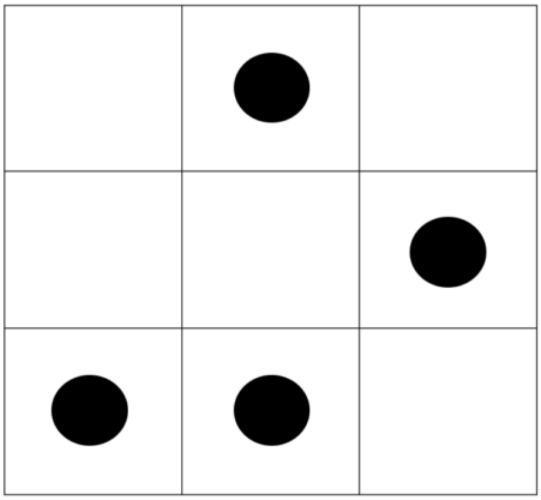

CPT Q5 – Question

Claire is 31 years old, single, outspoken and very bright. She majored in philosophy. As a student, she was deeply concerned with issues of discrimination and social justice, and also participated in anti-nuclear demonstrations.

Which is more probable?

- ○ Claire is a bank teller
- ○ Claire is a bank teller and is active in the feminist movement

CPT Q5 – Load test

Please choose the image that you saw right before answering the previous question.

○ 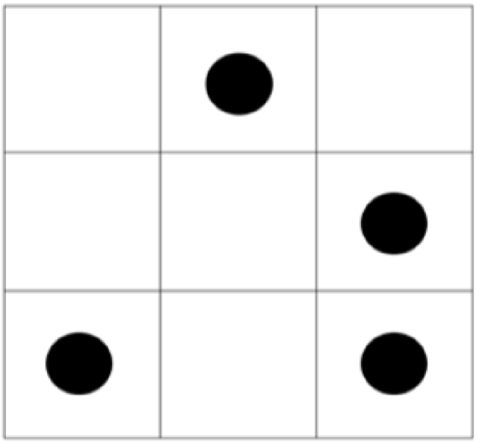

○ 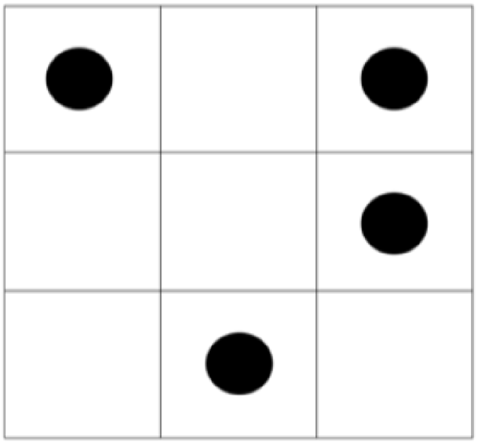

○ 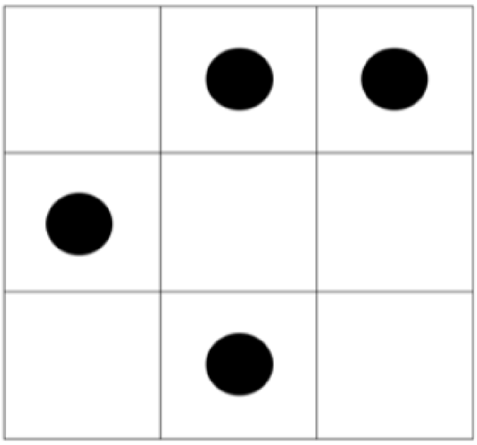

○ 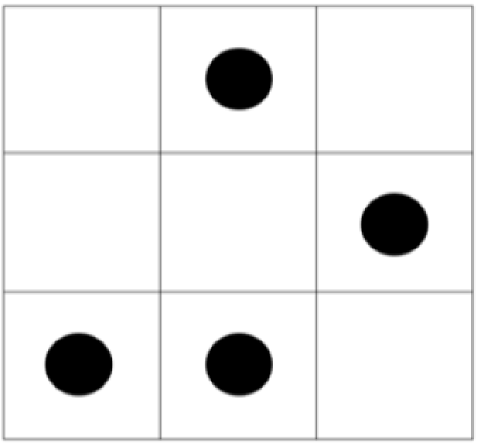

Very high cognitive load

Overview

Overview

There is only one task in this study: Task A.

You will earn £0.20 in addition to the participation fee of £0.42 if you closely follow all of the underlined instructions in this task.

Please continue to start Task A.

Task A

Task A

For this task, we ask you to answer five test questions in a row.

Your goal is to give as many correct answers as possible.

Task A continued

Task A

We ask that you memorize an image before answering each question.

The following screens will describe the memorization task in more detail.

Task A continued

An example of a matrix image is shown below.

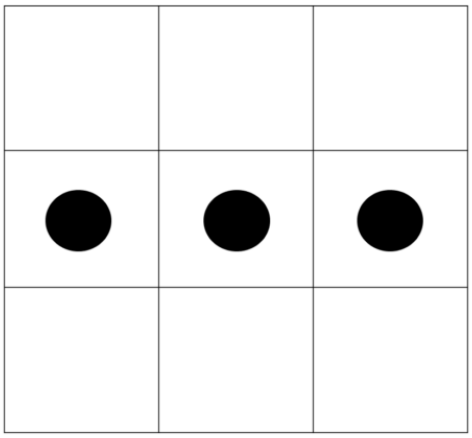

Task A continued

To be able to see the image, you will need to simultaneously press and keep pressing the "Escape" and "Backspace" keys on your keyboard. The position of these keys are shown below in red circles. You should use your left hand to press "Escape" and your right hand to press "Backspace".

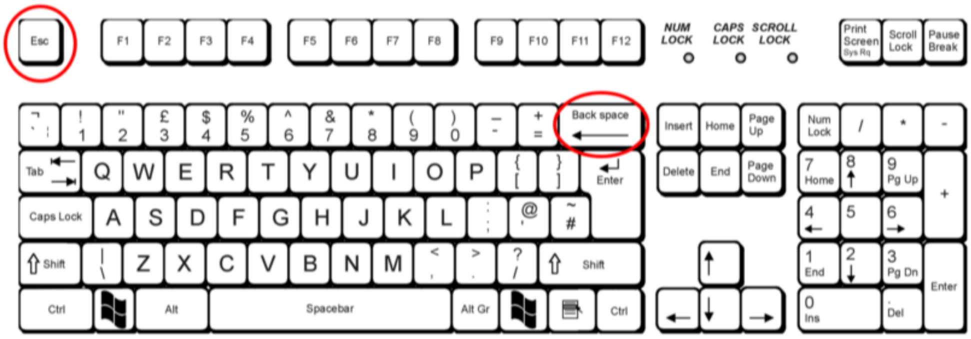

The image will be displayed for 5 seconds or until you stop pushing one or both buttons, whichever comes earlier.

You can practice viewing the image on the next screen.

Task A continued

This is a practice screen. To view the image, please push "Escape" and "Backspace" buttons at the same time. Keep pushing the two buttons until the next screen appears or until you memorize the position of the dots.

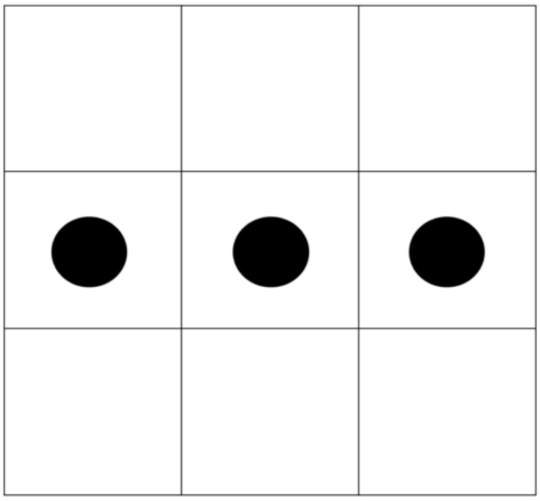

Task A continued

You need to memorize the displayed image before answering each question and keep this image in your mind while answering the question.

You will be asked to correctly identify the image after having answered the question.

You can click "Back" to practice viewing the image or click "Continue" to start Task A.

Note: CPT questions and answers were randomly presented, and the question text became visible two seconds before the answers.

CPT Q1 – Load

Please view the image by pushing "Escape" and "Backspace" buttons at the same time. You have at most 5 seconds to memorize the figure.

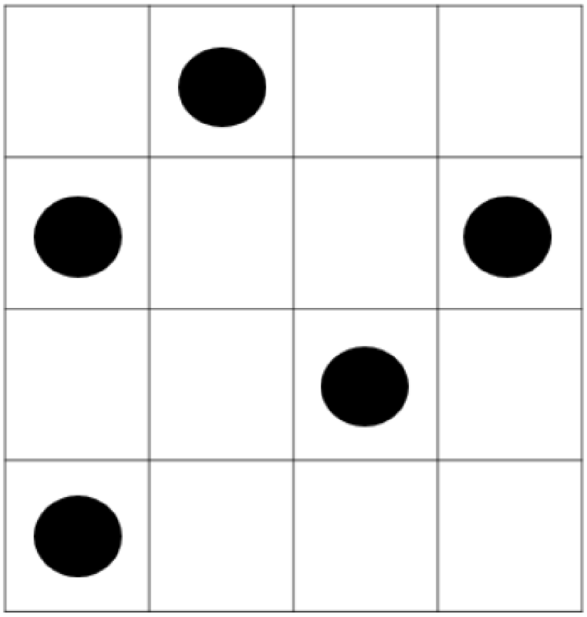

CPT Q1 - Question

A bat and a ball cost £1.10 in total. The bat costs £1.00 more than the ball. How much does the ball cost?

- ○ 10 pence
- ○ 9 pence
- ○ 1 pence
- ○ 5 pence

CPT Q1 – Load test

Please choose the image that you saw right before answering the previous question.

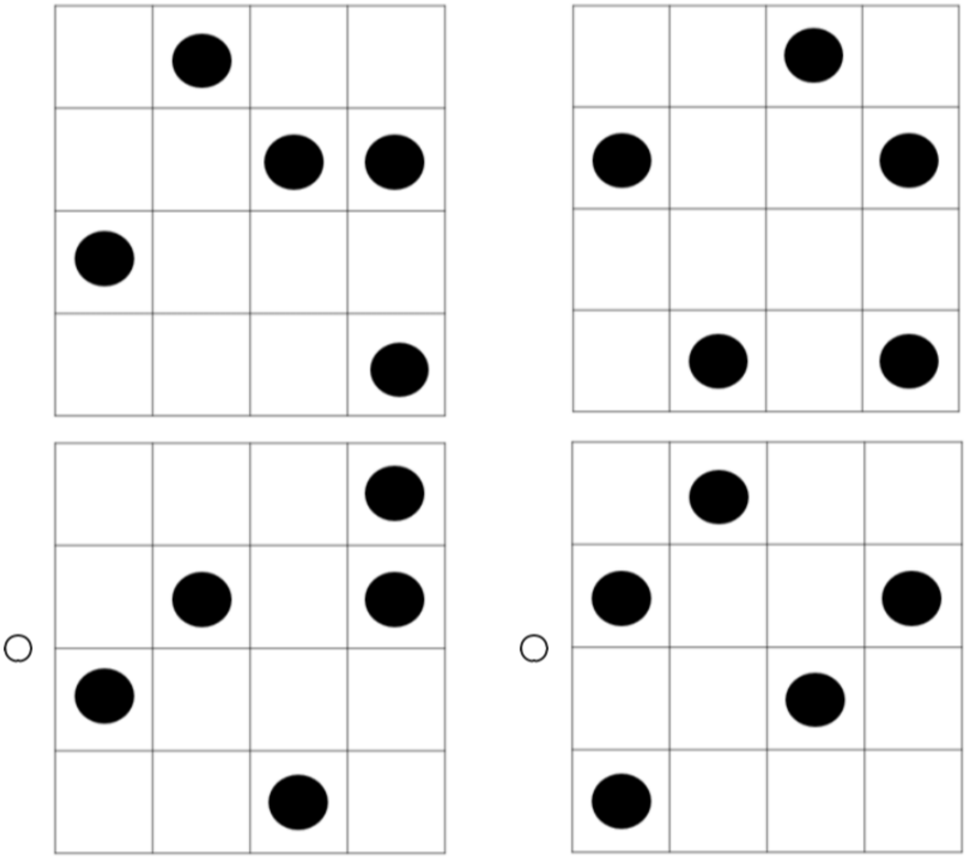

CPT Q2 – Load

Please view the image by pushing "Escape" and "Backspace" buttons at the same time.
You have at most 5 seconds to memorize the figure.

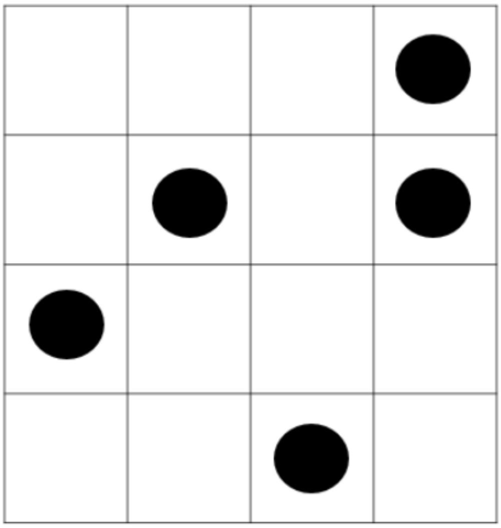

CPT Q2 – Question

If it takes 5 machines 5 minutes to make 5 widgets, how long would it take 100 machines to make 100 widgets?

- ◯ 500 minutes
- ◯ 100 minutes
- ◯ 20 minutes
- ◯ 5 minutes

CPT Q2 – Load test

Please choose the image that you saw right before answering the previous question.

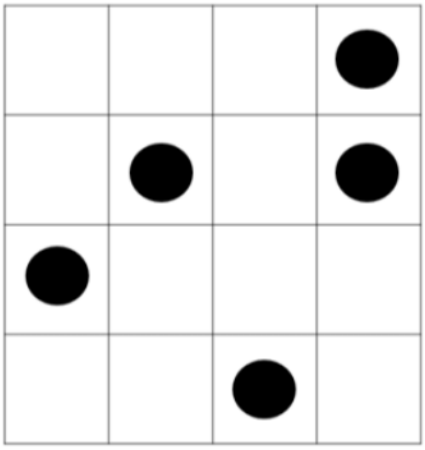

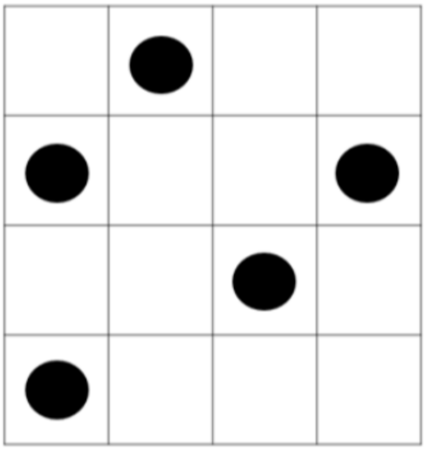

◯ 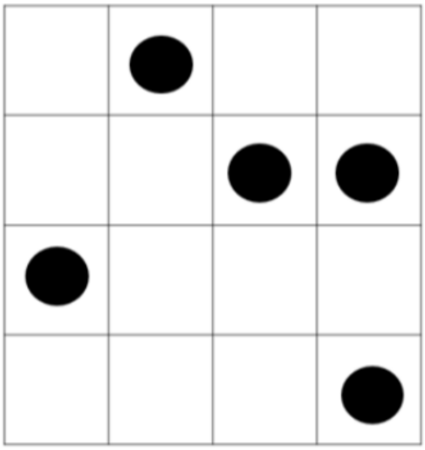

◯ 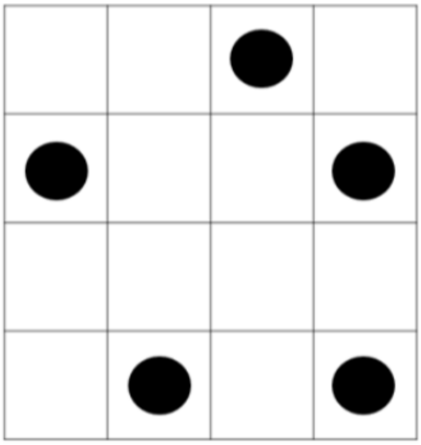

CPT Q3 – Load

Please view the image by pushing "Escape" and "Backspace" buttons at the same time. You have at most 5 seconds to memorize the figure.

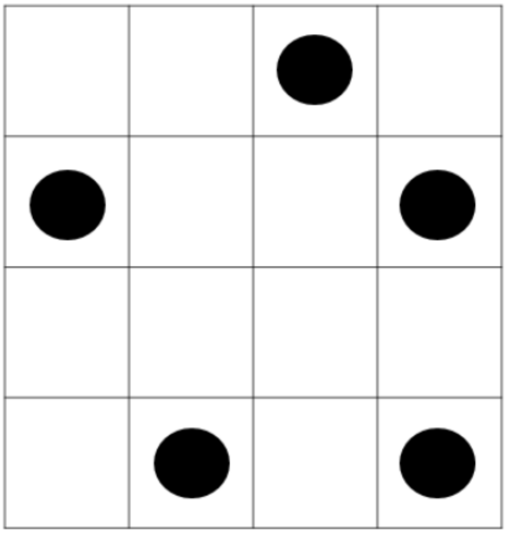

CPT Q3 – Question

In a lake, there is a patch of lily pads. Every day, the patch doubles in size. If it takes 48 days for the patch to cover the entire lake, how long would it take for the patch to cover half of the lake?

- ◯ 24 days
- ◯ 12 days
- ◯ 36 days
- ◯ 47 days

CPT Q3 – Load test

Please choose the image that you saw right before answering the previous question.

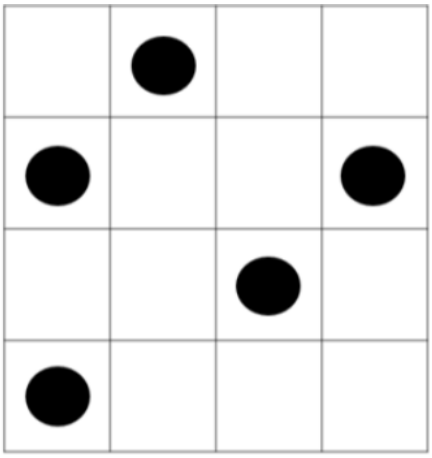

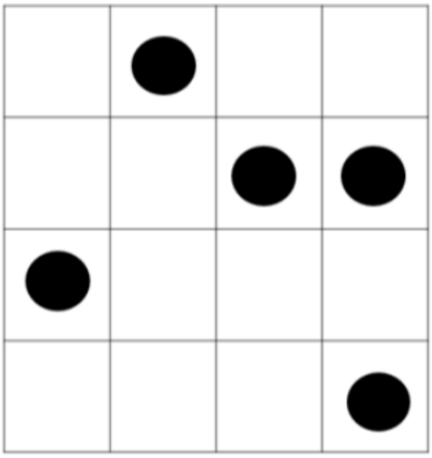

○ 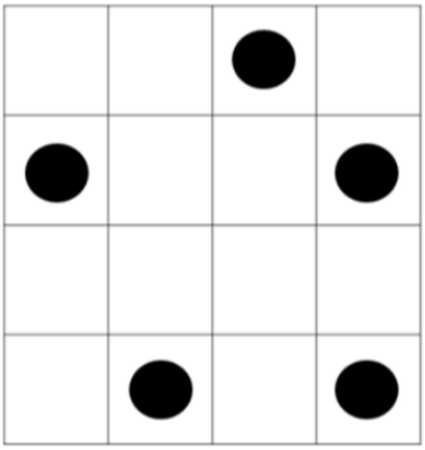 ○ 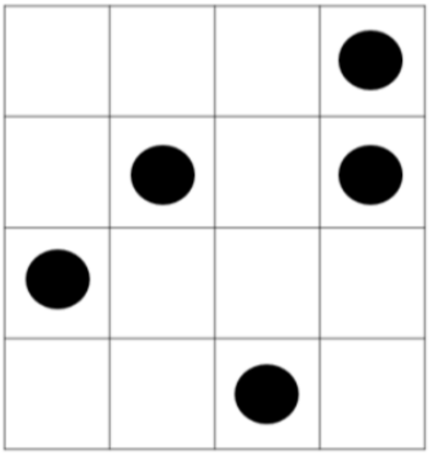

CPT Q4 – Load

Please view the image by pushing "Escape" and "Backspace" buttons at the same time.
You have at most 5 seconds to memorize the figure.

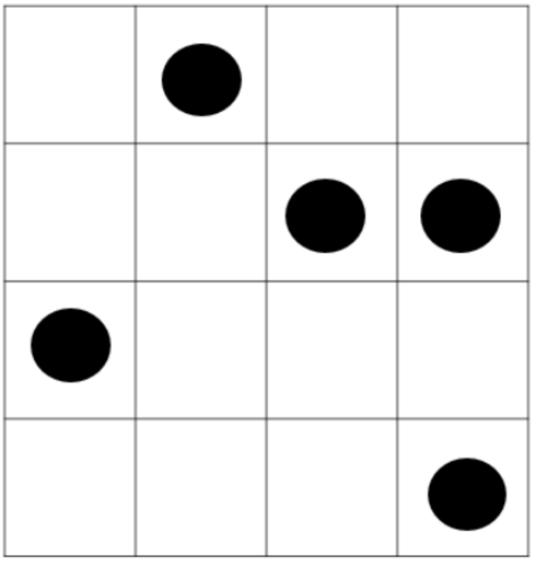

CPT Q4 – Question

- All living things need water.
- Roses need water.

If these two statements are true, can we conclude from them that roses are living things?

○ Yes
○ No

CPT Q4 – Load test

Please choose the image that you saw right before answering the previous question.

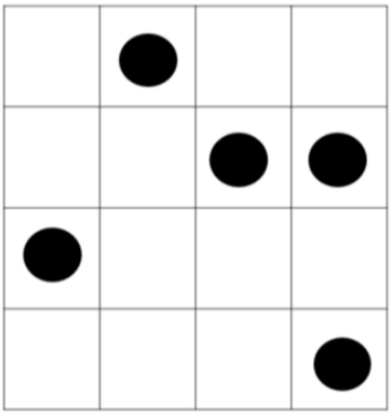

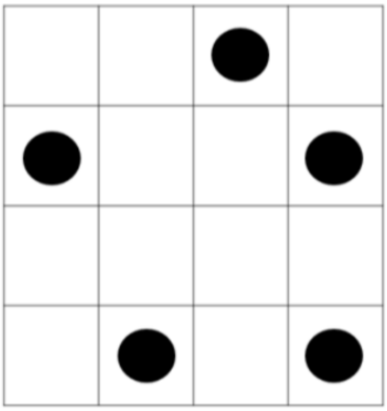

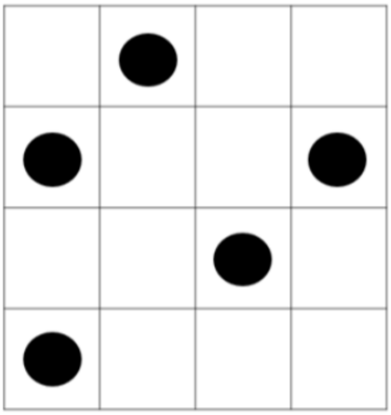

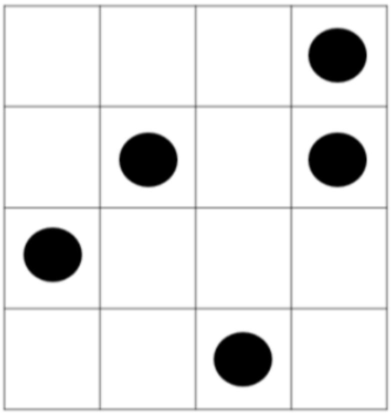

CPT Q5 – Load

Please view the image by pushing "Escape" and "Backspace" buttons at the same time.
You have at most 5 seconds to memorize the figure.

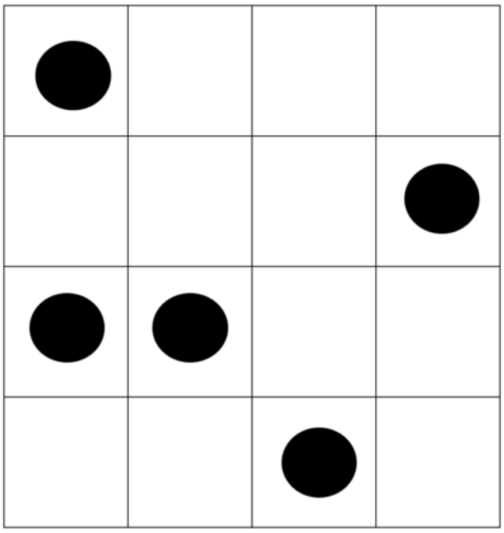

CPT Q5 – Question

Claire is 31 years old, single, outspoken and very bright. She majored in philosophy. As a student, she was deeply concerned with issues of discrimination and social justice, and also participated in anti-nuclear demonstrations.

Which is more probable?

- ○ Claire is a bank teller
- ○ Claire is a bank teller and is active in the feminist movement

CPT Q5 – Load test

Please choose the image that you saw right before answering the previous question.

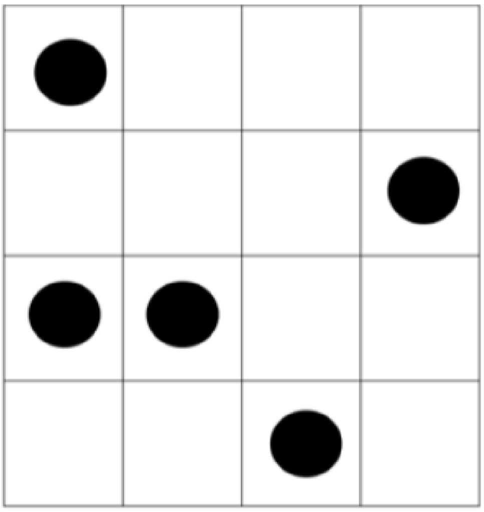

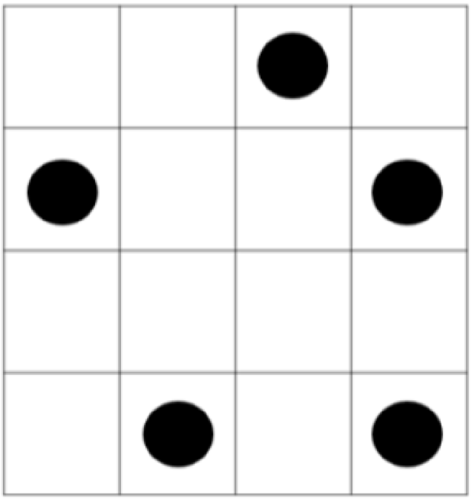

○ 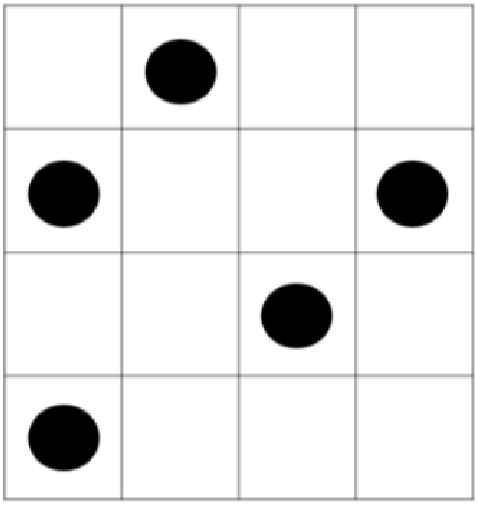

○ 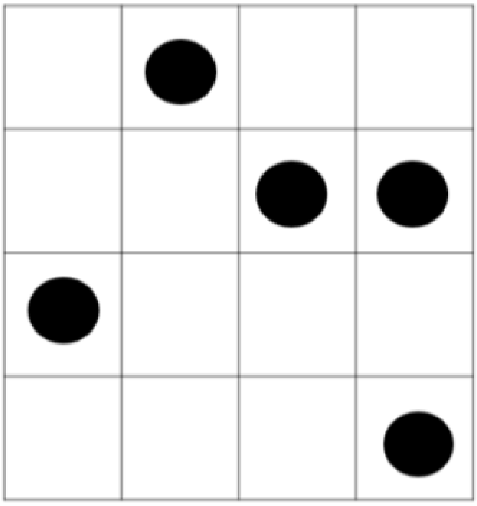

Short debiasing training

Overview

Overview

There are two tasks in this study: Task A and Task B.

You will earn £0.20 in addition to the participation fee of £0.42 if you closely follow all of the underlined instructions in both tasks.

Please continue to start Task A.

Task A

Task A

In this task, you will be asked to answer three questions in a row.

After you answer each question, we will provide you with the correct answer. Please read these correct answers carefully.

Task A continued

1. How many of each animal did Moses take on the ark?

Task A continued

1. How many of each animal did Moses take on the ark?

Correct Answer: "Noah had an ark, not Moses."

Task A continued

2. In a study, 1000 people were tested. Among the participants, there were 5 engineers and 995 lawyers. Jack is a randomly chosen participant in this study. Jack is 36 years old. He is not married and is somewhat introverted. He likes to spend his free time reading science fiction and writing computer programs. What is most likely?

○ Jack is a lawyer
○ Jack is an engineer

Task A continued

2. In a study, 1000 people were tested. Among the participants, there were 5 engineers and 995 lawyers. Jack is a randomly chosen participant in this study. Jack is 36 years old. He is not married and is somewhat introverted. He likes to spend his free time reading science fiction and writing computer programs. What is most likely?

Correct Answer: "Jack is a lawyer"

Task A continued

3. Which cause more human deaths, sharks or horses?

○ Sharks
○ Horses

Task A continued

3. Which cause more human deaths, sharks or horses?

Correct Answer: "Horses"

Task A continued

Conclusion: Our judgments are open to numerous biases. Therefore, it is very important to pause and reconsider our spontaneous reactions before making a decision.

In the next task, we ask you to <u>carefully reflect on each question by pausing and reconsidering your initial answer before submitting your decision.</u>

Task B

Task B

For this task, we ask you to answer five test questions in a row.

Your goal is to give as many correct answers as possible.

Note: CPT questions and answers were randomly presented, and the question text became visible two seconds before the answers.

CPT Q1

A bat and a ball cost £1.10 in total. The bat costs £1.00 more than the ball. How much does the ball cost?

Pause and reconsider your initial answer.

- ○ 10 pence
- ○ 9 pence
- ○ 1 pence
- ○ 5 pence

CPT Q2

If it takes 5 machines 5 minutes to make 5 widgets, how long would it take 100 machines to make 100 widgets?

Pause and reconsider your initial answer.

- ○ 500 minutes
- ○ 100 minutes
- ○ 20 minutes
- ○ 5 minutes

CPT Q3

In a lake, there is a patch of lily pads. Every day, the patch doubles in size. If it takes 48 days for the patch to cover the entire lake, how long would it take for the patch to cover half of the lake?

Pause and reconsider your initial answer.

- ○ 24 days
- ○ 12 days
- ○ 36 days
- ○ 47 days

CPT Q4

- All living things need water.
- Roses need water.

If these two statements are true, can we conclude from them that roses are living things?

Pause and reconsider your initial answer.

- ○ Yes
- ○ No

CPT Q5

Claire is 31 years old, single, outspoken and very bright. She majored in philosophy. As a student, she was deeply concerned with issues of discrimination and social justice, and also participated in anti-nuclear demonstrations.

Which is more probable?

Pause and reconsider your initial answer.

- ○ Claire is a bank teller
- ○ Claire is a bank teller and is active in the feminist movement

Long debiasing training

Overview

Overview

There are two tasks in this study: Task A and Task B.

You will earn £0.20 in addition to the participation fee of £0.42 if you closely follow all of the underlined instructions in both tasks.

Please continue to start Task A.

Task A

Task A

In this task, you will be asked to answer three questions in a row.

After you answer each question, we will provide you with an explanation of the correct answer. Please read these explanations carefully.

Task A continued

1. How many of each animal did Moses take on the ark?

Task A continued

1. How many of each animal did Moses take on the ark?

Correct Answer: "Noah had an ark, not Moses."

Explanation: Some people tend to think intuitively, using their "gut reaction". These people tend to give the answer "2" or "a pair" to this question. However, a more careful reading allows us to notice why this is a trick question because it says Moses's, *not* Noah's, ark. The correct answer is "Noah had an ark, not Moses." This question is used to measure the tendency to think intuitively, and those who resist their immediate response and reflect on the question generally answer it correctly.

Please continue to the second question.

Task A continued

2. In a study, 1000 people were tested. Among the participants, there were 5 engineers and 995 lawyers. Jack is a randomly chosen participant in this study. Jack is 36 years old. He is not married and is somewhat introverted. He likes to spend his free time reading science fiction and writing computer programs. What is most likely?

◯ Jack is a lawyer
◯ Jack is an engineer

Task A continued

2. In a study, 1000 people were tested. Among the participants, there were 5 engineers and 995 lawyers. Jack is a randomly chosen participant in this study. Jack is 36 years old. He is not married and is somewhat introverted. He likes to spend his free time reading science fiction and writing computer programs. What is most likely?

Correct Answer: “Jack is a lawyer”

Explanation: People frequently give the answer “Jack is an engineer” to this question, especially those who tend to rely on their intuitive responses. This is because the person described in the question activates in our minds the image of a *stereotypical* engineer. However, a careful rereading of the question makes clear that Jack was selected not based on who he is but rather chosen *randomly from one thousand people*. Therefore, the selected person has 99.5% (995 in 1000) chance of being a lawyer and only 0.5% (5 in 1000) chance of being an engineer. The question is designed to trick the reader by giving a stereotypical description of Jack, thereby leading them towards the intuitive response. Reflective thinkers, those who tend to *think twice* rather than answering with their “gut”, tend to answer this question correctly (i.e., “Jack is a lawyer” is the most likely scenario).

Please continue to the third question.

Task A continued

3. Which cause more human deaths, sharks or horses?

◯ Sharks
◯ Horses

Task A continued

3. Which cause more human deaths, sharks or horses?

Correct Answer: "Horses"

Explanation: Most people say "sharks" cause more human deaths than "horses", which is *false*. For example, according to a national database, there were 77 deaths related to horses, ponies or donkeys, whereas there were only 16 shark-related deaths in Australia between 2000 and 2010. So why do most people think that sharks are deadlier than horses?

When asked about the likelihood of an event, we often intuitively rely on *how easily we recall memories of such events rather than the actual frequency of these events*. Because deaths by shark attacks receive more media coverage than horse-related deaths, we are more likely to remember those episodes, which bias our judgments. This phenomenon is called the availability heuristic.

Please continue.

Task A continued

Task A

Please write a paragraph with four sentences (one sentence in each of the four cells below) that summarizes what you have learned in this task about thinking carefully.

1)

2)

3)

4)

Task A continued

Conclusion: Our judgments are open to numerous biases. Therefore, it is very important to pause and reconsider our spontaneous reactions before making a decision.

In the next task, we ask you to carefully reflect on each question by pausing and reconsidering your initial answer before submitting your decision.

Task B

Task B

For this task, we ask you to answer five test questions in a row.

Your goal is to give as many correct answers as possible.

Note: CPT questions and answers were randomly presented, and the question text became visible two seconds before the answers.

CPT Q1

A bat and a ball cost £1.10 in total. The bat costs £1.00 more than the ball. How much does the ball cost?

Pause and reconsider your initial answer.

- ○ 10 pence
- ○ 9 pence
- ○ 1 pence
- ○ 5 pence

CPT Q2

If it takes 5 machines 5 minutes to make 5 widgets, how long would it take 100 machines to make 100 widgets?

Pause and reconsider your initial answer.

- ◯ 500 minutes
- ◯ 100 minutes
- ◯ 20 minutes
- ◯ 5 minutes

CPT Q3

In a lake, there is a patch of lily pads. Every day, the patch doubles in size. If it takes 48 days for the patch to cover the entire lake, how long would it take for the patch to cover half of the lake?

Pause and reconsider your initial answer.

- ◯ 24 days
- ◯ 12 days
- ◯ 36 days
- ◯ 47 days

CPT Q4

- All living things need water.
- Roses need water.

If these two statements are true, can we conclude from them that roses are living things?

Pause and reconsider your initial answer.

- ◯ Yes
- ◯ No

CPT Q5

Claire is 31 years old, single, outspoken and very bright. She majored in philosophy. As a student, she was deeply concerned with issues of discrimination and social justice, and also participated in anti-nuclear demonstrations.

Which is more probable?

Pause and reconsider your initial answer.

- ◯ Claire is a bank teller
- ◯ Claire is a bank teller and is active in the feminist movement

Decision justification

Overview

Overview

There is only one task in this study: Task A.

You will earn £0.20 in addition to the participation fee of £0.42 if you closely follow all of the underlined instructions in this task.

Please continue to start Task A.

Task A

Task A

For this task, we ask you to answer five test questions in a row.

Your goal is to give as many correct answers as possible.

Task A continued

Task A

Please explain your answer to each question by writing a description of your reasoning in one sentence or more.

Note: CPT questions and answers were randomly presented, and the question text became visible two seconds before the answers.

CPT Q1

A bat and a ball cost £1.10 in total. The bat costs £1.00 more than the ball. How much does the ball cost?

Explain your reasoning.

- ◯ 10 pence
- ◯ 9 pence
- ◯ 1 pence
- ◯ 5 pence

Explanation:

CPT Q2

If it takes 5 machines 5 minutes to make 5 widgets, how long would it take 100 machines to make 100 widgets?

Explain your reasoning.

- ◯ 500 minutes
- ◯ 100 minutes
- ◯ 20 minutes
- ◯ 5 minutes

Explanation:

CPT Q3

In a lake, there is a patch of lily pads. Every day, the patch doubles in size. If it takes 48 days for the patch to cover the entire lake, how long would it take for the patch to cover half of the lake?

Explain your reasoning.

- ○ 24 days
- ○ 12 days
- ○ 36 days
- ○ 47 days

Explanation:

CPT Q4

- All living things need water.
- Roses need water.

If these two statements are true, can we conclude from them that roses are living things?

Explain your reasoning.

- ○ Yes
- ○ No

Explanation:

CPT Q5

Claire is 31 years old, single, outspoken and very bright. She majored in philosophy. As a student, she was deeply concerned with issues of discrimination and social justice, and also participated in anti-nuclear demonstrations.

Which is more probable?

Explain your reasoning.

- ◯ Claire is a bank teller
- ◯ Claire is a bank teller and is active in the feminist movement

Explanation:

Monetary incentives

Overview

Overview

There is only one task in this study: Task A.

You can earn up to £1.00 in addition to the participation fee of £0.42 depending on your decisions.

Please continue to start Task A.

Task A

Task A

For this task, we ask you to answer five test questions in a row.

Your goal is to give as many correct answers as possible.

Task A continued

Task A

You will earn £0.20 for each correct answer, up to a total of £1.

Note: CPT questions and answers were randomly presented, and the question text became visible two seconds before the answers.

CPT Q1

A bat and a ball cost £1.10 in total. The bat costs £1.00 more than the ball. How much does the ball cost?

- ○ 10 pence
- ○ 9 pence
- ○ 1 pence
- ○ 5 pence

CPT Q2

If it takes 5 machines 5 minutes to make 5 widgets, how long would it take 100 machines to make 100 widgets?

- ○ 500 minutes
- ○ 100 minutes
- ○ 20 minutes
- ○ 5 minutes

CPT Q3

In a lake, there is a patch of lily pads. Every day, the patch doubles in size. If it takes 48 days for the patch to cover the entire lake, how long would it take for the patch to cover half of the lake?

- ○ 24 days
- ○ 12 days
- ○ 36 days
- ○ 47 days

CPT Q4

- All living things need water.
- Roses need water.

If these two statements are true, can we conclude from them that roses are living things?

- ○ Yes
- ○ No

CPT Q5

Claire is 31 years old, single, outspoken and very bright. She majored in philosophy. As a student, she was deeply concerned with issues of discrimination and social justice, and also participated in anti-nuclear demonstrations.

Which is more probable?

- ○ Claire is a bank teller
- ○ Claire is a bank teller and is active in the feminist movement

Standard two-response

Overview

Overview

There are two tasks in this study: Task A and Task B.

You will earn £0.20 in addition to the participation fee of £0.42 if you closely follow all of the underlined instructions in both tasks.

Please continue to start Task A.

Task A

Task A

For this task, we ask you to answer five test questions in a row.

Your goal is to give as many correct answers as possible.

Task A continued

Task A

We ask that you answer each question within 5 seconds.

Please answer each question by quickly indicating your immediate response.

Note: CPT questions and answers were randomly presented, the question text and the counter (started counting and) became visible two seconds before the answers.

CPT Q1

A bat and a ball cost £1.10 in total. The bat costs £1.00 more than the ball. How much does the ball cost?

Be quick!

◯ 10 pence
◯ 9 pence
◯ 1 pence
◯ 5 pence

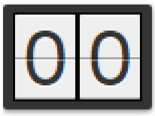

CPT Q2

If it takes 5 machines 5 minutes to make 5 widgets, how long would it take 100 machines to make 100 widgets?

Be quick!

◯ 500 minutes
◯ 100 minutes
◯ 20 minutes
◯ 5 minutes

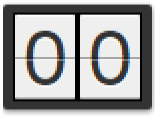

CPT Q3

In a lake, there is a patch of lily pads. Every day, the patch doubles in size. If it takes 48 days for the patch to cover the entire lake, how long would it take for the patch to cover half of the lake?

Be quick!

◯ 24 days
◯ 12 days
◯ 36 days
◯ 47 days

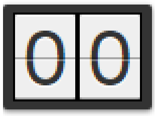

CPT Q4

- All living things need water.
- Roses need water.

If these two statements are true, can we conclude from them that roses are living things?

Be quick!

- ○ Yes
- ○ No

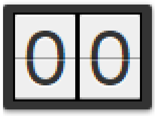

CPT Q5

Claire is 31 years old, single, outspoken and very bright. She majored in philosophy. As a student, she was deeply concerned with issues of discrimination and social justice, and also participated in anti-nuclear demonstrations.

Which is more probable?

Be quick!

- ○ Claire is a bank teller
- ○ Claire is a bank teller and is active in the feminist movement

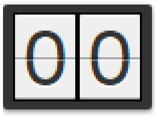

Task B

Task B

You are given a final opportunity to revise or confirm your answers to the test questions. You don't have to but you may choose to revise your initial response.

We ask that you think for at least 10 seconds before answering each question. Please answer each question after carefully considering your decision.

On each question, the "Continue" button will appear after 10 seconds.

Note: CPT questions and answers were randomly presented, the question text and the counter (started counting and) became visible two seconds before the answers and the "Continue" button appeared 10 seconds after the answers became visible. The answer given to each CPT question in Part A was displayed after the phrase "Your initial answer was…"

CPT Q1

A bat and a ball cost £1.10 in total. The bat costs £1.00 more than the ball. How much does the ball cost?

Your initial answer was

Carefully consider and either revise or confirm your answer.

- 10 pence
- 9 pence
- 1 pence
- 5 pence

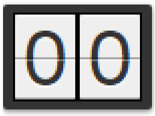

CPT Q2

If it takes 5 machines 5 minutes to make 5 widgets, how long would it take 100 machines to make 100 widgets?

Your initial answer was

Carefully consider and either revise or confirm your answer.

- 500 minutes
- 100 minutes
- 20 minutes
- 5 minutes

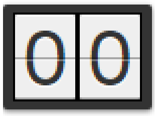

CPT Q3

In a lake, there is a patch of lily pads. Every day, the patch doubles in size. If it takes 48 days for the patch to cover the entire lake, how long would it take for the patch to cover half of the lake?

Your initial answer was

Carefully consider and either revise or confirm your answer.

- 24 days
- 12 days
- 36 days
- 47 days

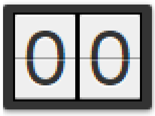

CPT Q4

- All living things need water.
- Roses need water.

If these two statements are true, can we conclude from them that roses are living things?

Your initial answer was

Carefully consider and either revise or confirm your answer.

○ Yes
○ No

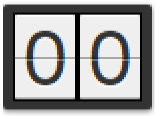

CPT Q5

Claire is 31 years old, single, outspoken and very bright. She majored in philosophy. As a student, she was deeply concerned with issues of discrimination and social justice, and also participated in anti-nuclear demonstrations.

Which is more probable?

Your initial answer was

Carefully consider and either revise or confirm your answer.

○ Claire is a bank teller
○ Claire is a bank teller and is active in the feminist movement

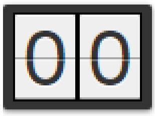

Modified two-response

Overview

Overview

There are two tasks in this study: Task A and Task B.

You will earn £0.20 in addition to the participation fee of £0.42 if you closely follow all of the underlined instructions in both tasks.

Please continue to start Task A.

Task A

Task A

For this task, we ask you to answer five test questions in a row.

Your goal is to give as many correct answers as possible.

Task A continued

Task A

We ask that you answer each question within 5 seconds.

Please answer each question by quickly indicating your immediate response.

Note: CPT questions and answers were randomly presented, the question text and the counter (started counting and) became visible two seconds before the answers.

CPT Q1

A bat and a ball cost £1.10 in total. The bat costs £1.00 more than the ball. How much does the ball cost?

Be quick!

- ◯ 10 pence
- ◯ 9 pence
- ◯ 1 pence
- ◯ 5 pence

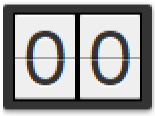

CPT Q2

If it takes 5 machines 5 minutes to make 5 widgets, how long would it take 100 machines to make 100 widgets?

Be quick!

- ◯ 500 minutes
- ◯ 100 minutes
- ◯ 20 minutes
- ◯ 5 minutes

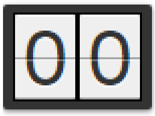

CPT Q3

In a lake, there is a patch of lily pads. Every day, the patch doubles in size. If it takes 48 days for the patch to cover the entire lake, how long would it take for the patch to cover half of the lake?

Be quick!

- ◯ 24 days
- ◯ 12 days
- ◯ 36 days
- ◯ 47 days

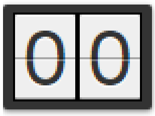

CPT Q4

- All living things need water.
- Roses need water.

If these two statements are true, can we conclude from them that roses are living things?

Be quick!

◯ Yes
◯ No

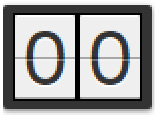

CPT Q5

Claire is 31 years old, single, outspoken and very bright. She majored in philosophy. As a student, she was deeply concerned with issues of discrimination and social justice, and also participated in anti-nuclear demonstrations.

Which is more probable?

Be quick!

◯ Claire is a bank teller
◯ Claire is a bank teller and is active in the feminist movement

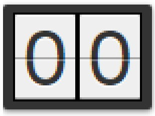

Task B

Task B

You are given a final opportunity to revise or confirm your answers to the test questions. You don't have to but you may choose to revise your initial response.

We ask that you think for at least 10 seconds before answering each question. Please answer each question after carefully considering your decision.

Also, please explain your answer to each question by writing a description of your reasoning in one sentence or more.

On each question, the "Continue" button will appear after 10 seconds.

Note: CPT questions and answers were randomly presented, the question text and the counter (started counting and) became visible two seconds before the answers and the "Continue" button appeared 10 seconds after the answers became visible. The answer given to each CPT question in Part A was displayed after the phrase "Your initial answer was…"

CPT Q1

A bat and a ball cost £1.10 in total. The bat costs £1.00 more than the ball. How much does the ball cost?

Your initial answer was

Carefully consider and either revise or confirm your answer. Explain your reasoning.

- ○ 10 pence
- ○ 9 pence
- ○ 1 pence
- ○ 5 pence

Explanation:

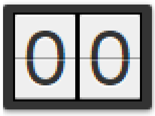

CPT Q2

If it takes 5 machines 5 minutes to make 5 widgets, how long would it take 100 machines to make 100 widgets?

Your initial answer was

Carefully consider and either revise or confirm your answer. Explain your reasoning.

- ◯ 500 minutes
- ◯ 100 minutes
- ◯ 20 minutes
- ◯ 5 minutes

Explanation:

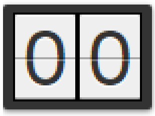

CPT Q3

In a lake, there is a patch of lily pads. Every day, the patch doubles in size. If it takes 48 days for the patch to cover the entire lake, how long would it take for the patch to cover half of the lake?

Your initial answer was

Carefully consider and either revise or confirm your answer. Explain your reasoning.

- ◯ 24 days
- ◯ 12 days
- ◯ 36 days
- ◯ 47 days

Explanation:

00

CPT Q4

- All living things need water.
- Roses need water.

If these two statements are true, can we conclude from them that roses are living things?

Your initial answer was

Carefully consider and either revise or confirm your answer. Explain your reasoning.

- ○ Yes
- ○ No

Explanation:

00

CPT Q5

Claire is 31 years old, single, outspoken and very bright. She majored in philosophy. As a student, she was deeply concerned with issues of discrimination and social justice, and also participated in anti-nuclear demonstrations.

Which is more probable?

Your initial answer was

Carefully consider and either revise or confirm your answer. Explain your reasoning.

- ○ Claire is a bank teller
- ○ Claire is a bank teller and is active in the feminist movement

Explanation:

00

Self-reported reflection

Please complete this section about how you answered the five previous test questions.

To what extent did you rely on your feelings or gut-reactions when making your decisions?

not at all 0	1	2	3	4	5	6	7	8	9	very much 10
○	○	○	○	○	○	○	○	○	○	○

To what extent did you rely on reason when making your decisions?

not at all 0	1	2	3	4	5	6	7	8	9	very much 10
○	○	○	○	○	○	○	○	○	○	○
